# Navigating the Landscape of Exosomal microRNAs: Charting Their Pivotal Role as Biomarkers in Hematological Malignancies

**DOI:** 10.3390/ncrna11050064

**Published:** 2025-08-31

**Authors:** Manlio Fazio, Fabio Stagno, Giuseppa Penna, Giuseppe Mirabile, Alessandro Allegra

**Affiliations:** Division of Hematology, Department of Human Pathology in Adulthood and Childhood “Gaetano Barresi”, University of Messina, Via Consolare Valeria, 98125 Messina, Italy; manliofazio@hotmail.it (M.F.); giusypenna1@gmail.com (G.P.); giuseppe.mirabile@polime.it (G.M.); aallegra@unime.it (A.A.)

**Keywords:** extracellular vesicles, microRNA, intercellular communication, tumor-derived exosomes, hematological malignancies, microenvironment, biomarkers, liquid biopsy, target therapy

## Abstract

Under physiological and pathological conditions, all cells release extracellular vesicles named exosomes, which act as transporters of lipidic, protein, and genetic material from parent to recipient cells. Neoplastic cells can secrete higher number of exosomes to exert pro-tumoral effects such as microenvironmental changes, disease progression, immunosuppression and drug-resistance. This holds true for both organ-specific cancers and hematologic malignancies. One of the most important components of exosomal cargo are microRNAs which can mediate all the abovementioned effects. More specifically, microRNAs are small non-coding RNAs, routinely detected through quantitative real-time PCR, which act as translational suppressors by regulating protein-coding genes. Considering their high stability in all body fluids and viability in circulation, research is currently focusing on this type of RNAs for the so called “liquid biopsy”, a non-invasive tool for disease diagnosis and longitudinal monitoring. However, several issues remain to be solved including the lack of standardized protocols for exosome isolation and miRNA detection. Starting with this premise, our review aims to provide a wide description of the known microRNA panels employed in the prominent hematological malignancies, which will hopefully redefine the approach to these very challenging diseases in the near future.

## 1. Introduction

### General Considerations on Exosomes

Extracellular vesicles (EVs) are universally recognized as vehicles for the intercellular horizontal transfer of molecular signals and genes [1]. They were first described in 1960s through electron microscopy as 20–50 nm-sized vesicles carrying tissue factor (TF) in human platelet-free plasma [2]. They were also observed in tumor cell cultures and initially considered as “cellular waste” [3]. It is now established that EVs represent a complex system of short and long-distance delivery of cellular contents which is shared by all body districts (including hematopoietic and immune systems) [1]. At present, extracellular vesicles have been categorized into three main groups according to their size: (1) micro-vesicles (MVs) (also called ectosomes, shedding vesicles, microparticles) formed by outward budding from the plasma membrane (100–1000 nm); (2) exosomes, which are smaller than MVs (30–100 nm); and (3) apoptotic bodies (ABs), which are large clumps of material (1000–5000 nm) originating from cells undergoing apoptosis [4]. Exosomes present a unique origin and are secreted by many types of cells into various biological fluids (serum, plasma, urine, ascites, cerebrospinal fluid) [5]. They originate from multivesicular bodies (MVBs) located in the cellular endosomal compartment and are released externally after MVBs fusion with cell membrane. For this reason, exosomes present some endosome-associated proteins on their surface (such as TSG101 and ALIX) [6]. Exosomes also have a unique cargo since they carry cytosol components and various RNAs and DNAs [7]. For these reasons, these vesicles are involved in numerous physiological and pathological processes. The former includes tissue differentiation and repair, coagulation, autoimmunity, inflammation, and hematopoiesis [8], while the latter includes tumor expansion and dissemination, and drug-resistance [9]. In this regard, studies confirm that neoplastic cells secrete higher quantities of exosomes than healthy cells [10] and this event occurs in both solid and hematologic malignancies. These tumor-derived exosomes (TEXs) carry oncogenic proteins which are responsible for tumor growth [11]. Therefore, it has been suggested to employ EVs both as new promising, non-invasive biomarkers for tumor diagnosis and as potential therapeutic targets. As previously specified, exosomes may contain various RNAs, such as microRNAs (miRNAs). The first miRNA was described in 1993 in a nematode (*Caenorhabditis elegans*) [12]. In 2000s, another miRNA (Lin-4) was discovered in *C. elegans*. Seven years later, in 2007, Valadi et al. provided the first evidence that exosomes shuttled miRNAs into acceptor cells [7]. MiRNAs are small (19–25 nucleotides), non-coding RNA molecules whose function is to regulate post-transcriptional gene expression by degrading or suppressing their target messenger-RNA (mRNA) [13]. More specifically, miRNAs can affect the expression of one-third of human genes. This is because they do not require a complete pairing with the end sequences of 3′ untranslated regions (3′UTRs)-mRNAs. In particular, the selection of a target mRNA occurs by matching just six or seven nucleotides [14]. Quantitative real-time polymerase chain reaction (qRT-PCR) is the most frequently used method for the detection and quantification of circulating miRNAs [15]; other standard methods are small RNA sequencing and microarray. In organ-specific and hematologic malignancies, the microRNAs landscape depends on the transcriptome of the parental neoplastic cells and orchestrates a series of pro-tumoral effects such as normal cells malignant transformation, tumor expansion, remodeling of the microenvironmental niche, tumor relapse, and refractoriness to chemo-immunotherapy. For example, Feng et al. found that an increased expression of miR-99a-5p and miR-125-5p in plasma exosomes of diffuse large B-cell lymphoma (DLBCL) is associated with chemoresistance and a more unfavorable outcome [16]. Therefore, miRNAs appear as good candidates to be used for the classification and clustering of onco-hematologic diseases as well for diagnosis, prognosis definition, and prediction of progression or therapy response. In particular, miRNAs show some very convenient characteristics such as stability in circulation and diffusion in a wide range of biological samples (including metastatic tissue) [17]. Employing specific and standardized miRNA panels would allow an earlier detection of these pathological conditions in a pre-symptomatic stage and, most importantly, would allow us to perform the so-called liquid biopsy. The term “liquid biopsy” identifies a minimally invasive, repeatable, and highly informative diagnostic tool capable of tracking disease onset, progression, and therapeutic response in real time through the analysis of circulating biomarkers (tumor-derived components). Apart from peripheral blood, this technique can also be performed by using samples taken from other body fluids such as plasma, serum, cerebrospinal fluid, and urine. In addition, by characterizing these biomarkers, it would be possible to understand which of them could eventually be used to design even a future target therapy. Starting from this brief excursus, this review aims at providing a holistic vision on the biology and functions of exosomes and exosomal miRNAs in a wide spectrum of hematologic malignancies. Furthermore, it aims at describing the crosstalk between neoplastic cells and the BM microenvironment, describing potential mechanisms of disease resistance and relapse and exploring how miRNAs could serve as effective biomarkers of hematological neoplasms. To ensure a comprehensive and up-to-date synthesis of the literature, we performed a systematic search of multiple electronic databases, including PubMed/MEDLINE, Scopus, and Google Scholar, covering publications from January 2000 to June 2025. The search strategy combined controlled vocabulary (medical subject headings terms) and free-text keywords related to exosomes, extracellular vesicles, microRNA, hematological malignancies, liquid biopsy, diagnostic biomarkers, and individual disease terms (e.g., acute myeloid leukemia, multiple myeloma, chronic lymphocytic leukemia). Boolean operators (“AND”, “OR”), truncation, and proximity operators were applied to refine results and maximize retrieval of relevant studies. No language restrictions were applied, but priority was given to peer-reviewed articles, systematic reviews, and high-impact original research. Additionally, references from identified articles were hand-searched to capture seminal studies not retrieved in the initial search. Authors independently screened titles and abstracts, followed by full-text evaluation to include studies addressing the role of exosomal microRNAs in hematological malignancies.

## 2. Biogenesis of Extracellular Vesicles and Their RNA Cargo

### 2.1. Exosomes

**Figure 1:**
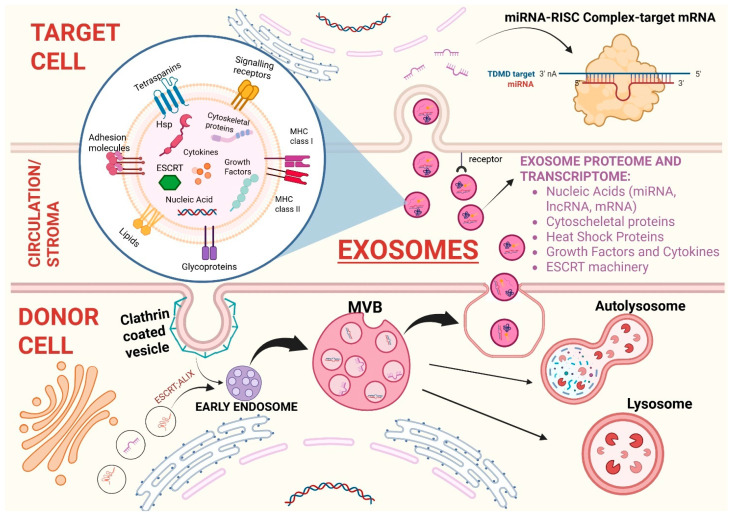
Biogenesis of exosomes and intercellular communication via extracellular vesicles. Biogenesis of exosomes from the formation of the early exosome after the invagination of the cellular membrane to multivesicular bodies (MVBs) that contain intraluminal vesicles (ILVs). MVBs can either fuse again with the cellular membrane and cause exosome exocytosis or fuse with lysosomes to be degraded in order to recycle cellular components and regulate signaling pathways. Once released in circulation/stromal microenvironment, exosomes protect their cargo from enzyme-mediated degradation and safely reach target cells. The magnified image at the center shows the exosome structure with its proteome and transcriptome (specified in the bullet point list on the right). Exosomes also comprise lipids such as cholesterol, ceramides, sphingomyelin, gangliosides, and phosphatidylserine. Once the recipient cell is reached, exosomes are internalized and bind with target mRNA determining its degradation or translational repression. ABBREVIATIONS: ESCR (endosomal sorting complexes required for transport); ALIX (ALG-2-interacting Protein X); MVBs (multivesicular bodies); miRNA (micro-RNA); lncRNA (long non-coding RNA); mRNA (messenger-RNA); Hsp (heat shock protein); TDMD (target-directed miRNA degradation); RISC (RNA-induced silencing complex).

The first step of exosome biogenesis is the formation of an early endosome due to the invagination of the cellular membrane of the parent cell. Therefore, the endosome membrane contains elements derived from plasma membrane such as clathrin and glycoproteins. Then, the endosomal membrane generates intraluminal vesicles which contain cytosolic components (nucleic acids, soluble factors, nucleoproteins, enzymes, and other cytosolic molecules). These formations are called multivesicular bodies (MVBs). Two elements that play a crucial role in MVBs biogenesis are the following: (1) the Endosomal Sorting Complex Required for Transport (ESCRT), which is a family of proteins whose function is sorting ubiquitinated proteins into the lumen of MVBs; (2) the Apoptosis-linked protein gene 2-interacting protein X (ALIX), which acts as an ESCRT-associated protein. Lately, MVBs fuse again with plasma membrane to be secreted externally in an ATP-dependent manner as virus-size membranous vesicles. The outward budding of the micro-vesicle population is followed by a fission event that in many ways resembles the abscission step in cytokinesis [18]. Once secreted in the circulation by parent cells, these vesicles travel directly to a specific recipient cell to deliver molecules and genetic information which can lead to phenotypic and functional changes [4]. Therefore, the information dispatched by EVs closely depends on the program of the parental cell and the vesicular structure guarantees that the molecular cargo will be delivered to the final target cells in its original state, without being degraded by extracellular enzymes. Focusing on the content of EVs, it has been demonstrated that these vesicles are able to carry various types of RNAs, including mRNA, miRNA, and other small non-coding RNA species such as RNA transcripts overlapping with protein coding regions, repeat sequences, structural RNAs, tRNA fragments, vault RNA, Y RNA, and small interfering RNAs. All these genetic materials are then translated into proteins by target cells [19]. When EVs reach predesigned target cells, they can be internalized via endocytosis/phagocytosis or membrane fusion and transfer biologically active molecules. On the other hand, they can employ activating or inhibitory molecules distributed on their membrane to bind specific receptors on target cells and trigger signaling pathways which can determine either cellular activation or suppression [20]. In Figure 1 the various steps of the exosome cycle are summarized, from biogenesis to internalization in the recipient cell. Biogenesis and mechanism of interaction between exosomes and malignant tumor cells differ from the interaction between exosomes and normal cells. More specifically, the tumor microenvironment is acidic, and the membrane of exosome released at low PH is characterized by higher rigidity and sphingomyelin/ganglioside GM3 content, which increases their fusion efficiency with cancer cells [20,21].

### 2.2. Tumor Derived Exosomes (TEXs)

Body fluids of cancer patients contain both tumor-derived and non-tumor-derived exosomes; and electron microscopy (EM) analysis does not show particular differences between these two species of exosomes. However, by using Immuno-EM, it is possible to demonstrate that TEXs are characterized by the presence of specific membrane molecules, such as FasL or glypican-1 [22]. The molecular content of TEXs partly resembles that of the parent cell cytosol and includes several RNA species, proteins, lipids, glycans, tumor-associated antigens (TAAs) and components of cellular signaling pathways such as β-catenin, WNT, and/or Notch [23]. Therefore, the molecular signature carried by TEXs varies according to the primary tumor cell lines [24]. In order to chart the content of TEXs isolated from supernatants of tumor cell lines, various methods can be employed including Western blots, immune arrays, and mass spectrometry [25]. TEXs distribute throughout all body fluids contributing to a communication network between tumor and host cells, but also between tumor cells themselves. Once the recipient cells are reached, TEXs are internalized by fusion, phagocytosis or endocytosis, and can either be directed to lysosomes for degradation or incorporated into the cellular machinery to initiate recipient cell reprogramming [26]. TEXs may also employ both autocrine and juxtacrine signaling to communicate with cells. All changes induced by TEXs in recipient cells are entirely tumor-driven and aim at favoring disease progression and metastasis by delivering depleted growth receptors, ectoenzymes, or factors that sustains tumor growth [27]. Another important aspect is the effect of TEXs on the microenvironment. In other words, TEXs are able to remodulate stromal cell functions and promote neo-angiogenesis. Moreover, TEXs can alter both innate and adaptive immune response through several mechanisms including the following: delivering immunosuppressive molecules to T lymphocytes and natural killer (NK) cells [28,29], promoting differentiation of regulatory T-cells (Tregs), myeloid-derived suppressor cells (MDSCs), and regulatory B-cells (Bregs) [28]. On the other hand, TEXs may also mediate the opposite effect, that is inducing anti-tumor immunity, by delivering TAAs to dendritic cells (DCs) or presenting these antigens to T lymphocytes [30]. Tumor derived exosomes largely contribute to the whole biological and clinical course of hematological malignancies from development to progression. In a study carried out by Caivano et al., the exosome counts in patients affected by chronic lymphocytic leukemia (CLL), non-Hodgkin’s lymphoma (NHL), Waldenstrom’s macroglobulinemia (WM), Hodgkin’s lymphoma (HL), multiple myeloma (MM), acute myeloid leukemia (AML), myeloproliferative neoplasms (MPNs), and myelodysplastic syndromes (MDS) were found significantly higher than normal. All these EVs contain specific TAAs levels (CD19 in B-cell neoplasms, CD38 in MM, CD33 in myeloid tumors, and CD30 in HL), which also correlate with clinical features [31]. Taking AML as an example, in patients with de novo AML, plasma is enriched with CD34+, CD33+, and CD117+ exosomes. Other proteins such as MHC molecules, adhesion proteins, membrane transporters or cytoskeletal components are also present. After induction chemotherapy, exosomes levels tend to decrease. This phenomenon reflects the reduction in exosome-producing blasts in BM. Therefore, AML-derived exosomes may serve as an indicator of therapy response.

### 2.3. MIRNAs

**Figure 2:**
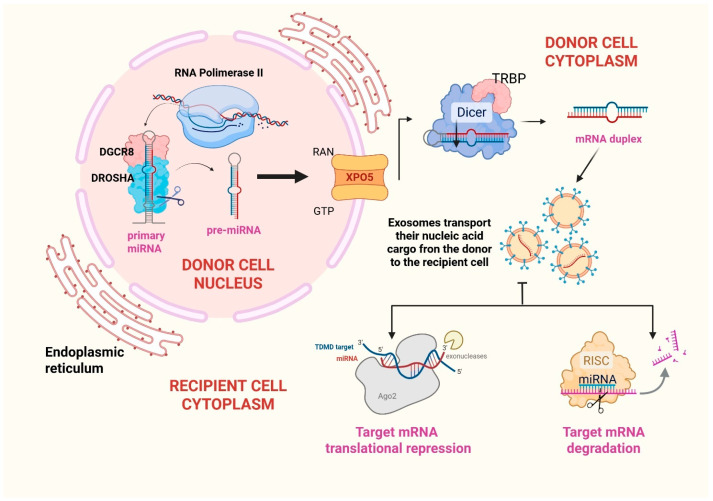
Micro-RNA biogenesis. MicroRNA genes are predominantly transcribed by RNA polymerase II, generating large primary transcripts known as pri-miRNAs. These pri-miRNAs undergo initial processing in the nucleus by the “microprocessor complex”, which comprises the RNA-binding protein DGCR8 and the RNase III enzyme Drosha. This cleavage yields a ~80-nucleotide hairpin precursor termed pre-miRNA. The pre-miRNA is subsequently exported to the cytoplasm via the Ran-GTP/Exportin-5 transport system. In the cytoplasm, the RNase III enzyme Dicer further processes the pre-miRNA into a ~20–22-nucleotide duplex. The image shows that, upon strand separation, the mature miRNA is incorporated into exosomes and conveyed to recipient cells. Once in the recipient cell, miRNAs are loaded in the RNA-induced silencing complex in order to target messenger-RNAs and lead to gene silencing. ABBREVIATIONS: RNA (ribonucleic acid); DGCR8 (DiGeorge syndrome critical region 8); XPO5 (Exportin-5); RAN (RAs-related nuclear protein); GTP (guanosine triphosphate); TRBP (trans-activation response RNA-binding protein); miRNA (micro-RNA); TDMD (target-directed miRNA degradation); RISC (RNA-induced silencing complex).

Micro-RNAs are a group of evolutionarily conserved, single-stranded, around 22-nucleotides long, regulatory, non-coding RNA (ncRNAs), which bind to the 3′UTR of target mRNAs and regulate gene expression at a post-translational level [32]. Furthermore, they transmit genetic materials from parent to recipient cells and regulate various pathways and processes (such as apoptosis [33], hematopoiesis [34], angiogenesis [35], metastasis [36]). Most of the genes responsible for miRNAs biogenesis are located in fragile chromosomal regions of the genome which are associated with cancers [37]. This further proves that miRNAs play an important role in the development of neoplastic diseases. The biogenesis of miRNAs consists of several steps. The first step occurs in the nucleus, where miRNA genes are transcribed into primary transcripts (pri-mRNA) by RNA polymerase II. These transcripts may present hairpin-like structures, which are subsequently processed and lead to the formation of 80-nucleotide-long stem-loop precursor miRNAs. Subsequently, Drosha ribonuclease III and the microprocessor complex subunit DGCR8 modify these precursors into pre-miRNAs. Then, miRNAs are exported from nucleus to cytoplasm by exportin-5 where RNase III Dicer [and its cofactor, trans-activation response (TAR) RNA-binding protein (TRBP)] further cleaves pre-miRNA. The guide strand of the miRNA is incorporated into the RNA-induced silencing complex (RISC), while the other strand is rapidly degraded. The miRNA-RISC then binds to the 3′UTR of target mRNA, specifically targeting those binding sites which are complementary to the guide strand. This interaction can lead to various silencing mechanisms. More specifically, the degree of miRNA-mRNA complementarity determines whether the miRNA will induce translational repression or mRNA degradation. When the complementarity is partial, the miRNA can inhibit translation. When the complementarity is strong, the miRNA can cause mRNA cleavage and degradation [38]. These processes are summarized in Figure 2.

## 3. Bioengineered Exosomes for miRNA Targeted Delivery and Related Therapeutic Prospectives

Exosomes are emerging as promising therapeutic agents and drug delivery vehicles in cancer treatment due to their natural ability to enter target cells, evade immune clearance, deliver functional payloads like miRNA, siRNA, chemotherapeutics, and proteins, and overcoming biological barriers such as the blood–brain barrier (BBB) and gastrointestinal tract. Some studies have shown that the co-administration of small functional RNAs and anticancer drugs via engineered exosomes allows overcoming drug resistance and represents an innovative treatment strategy for oncological diseases [39]. Compared to synthetic carriers such as liposomes, exosomes demonstrate superior biocompatibility and systemic retention, protecting their RNA cargo from degradation. Early studies report minimal toxicity, encouraging further development of exosome-based therapies for hematological and solid malignancies [40]. The employment of exosomes for drug delivery involves manifold passages. Exosomes isolation relies on methods such as ultracentrifugation (gold standard for high purity), precipitation techniques, size-based filtration, microfluidics, and immune-affinity capture. Each method balances yield, purity, and structural integrity, with emerging three-dimensional (3D) culture systems enhancing production efficiency. Tangential flow filtration (TFF) combined with 3D culture has increased yields up to 140-fold compared to 2D systems [41,42]. The advent of exosome-based drug delivery systems has prompted extensive investigation into efficient cargo loading techniques. Among these, pre-secretory (preloading) and post-secretory (postloading) strategies represent the two primary methodologies for engineering exosomes with therapeutic agents, particularly non-coding RNAs (ncRNAs) such as miRNAs, siRNAs, and antisense oligonucleotides (ASOs) [43,44]. Pre-secretory loading exploits the endogenous biogenesis pathway of exosomes to package therapeutic ncRNAs during vesicle formation within donor cells. Typically, this is achieved by transfecting or genetically engineering parent cells to overexpress the desired RNA molecules or RNA-binding proteins. As the MVBs mature and fuse with the plasma membrane, the engineered cargo is naturally sorted into the intraluminal vesicles (ILVs), which are subsequently released as exosomes [45]. This approach ensures high encapsulation efficiency and preserves the structural integrity of exosomal membranes. For instance, HEK293T cells transfected with let-7a precursors produced exosomes enriched with functional let-7a, effectively inhibiting breast cancer cell proliferation upon delivery [46]. Similarly, γδ T-cells engineered to overexpress miR-138 yielded exosomes with potent antitumor activity in oral squamous cell carcinoma models [47]. Another notable strategy involves the fusion of targeting ligands to exosomal membrane proteins such as lysosome-associated membrane glycoprotein 2b (Lamp2b). Bellavia et al. designed a Lamp2b-IL3 fusion protein enabling exosomes to specifically target CML blasts expressing IL3 receptor, while simultaneously packaging siBCR-ABL for gene silencing. Such pre-modification of donor cells enhances the precision of exosomal delivery [48]. However, pre-secretory loading is constrained by the cellular capacity for RNA packaging, potential cytotoxicity of transfection reagents, and variability in cargo sorting efficiency [45]. Post-secretory loading involves the manipulation of purified exosomes to introduce therapeutic ncRNAs after their secretion from donor cells. This method bypasses the need for donor cell genetic modification and enables precise control over the cargo composition [45]. Electroporation is the most widely used postloading technique for introducing siRNAs or miRNAs into exosomes. It has been demonstrated a successful delivery of TPD52-targeting siRNA into *HER2*-positive breast cancer cells using electroporated HEK293T-derived exosomes, achieving a 70% knockdown in gene expression [49]. Exosome-encapsulated CRISPR-Cas9 systems (often loaded via electroporation) have demonstrated significant potential in leukemia therapy [50]. Despite its popularity, electroporation may induce siRNA aggregation and exosome membrane destabilization, potentially reducing functional delivery. Alternative techniques such as sonication [51], extrusion, and saponin-mediated permeabilization [52] have been explored to enhance loading efficiency. Co-incubation, a milder approach, allows hydrophobic small molecules like curcumin to passively diffuse into exosomes, though its applicability to large ncRNAs remains limited [53]. In conclusion, both pre- and post-secretory strategies contribute to the evolving landscape of exosome engineering for ncRNA therapeutics. In the future, adopting a hybrid approach that combines both strategies would provide synergistic benefits.

## 4. Examining the Exosomal Mirnome in Hematological Malignancies

Recent investigations have substantially deepened our understanding of the critical role exosomes play in the early diagnosis, prognostication, and surveillance of hematological malignancies. For example, another type of non-coding RNA that can be contained in exosomes is vault RNA (vtRNA). Vaults are the largest ribonucleoprotein particles found in eukaryotic cells and are characterized by the major vault protein (MVP), two minor vault proteins (VPARP and Tep1), and a variety of small untranslated RNA molecules known as vault RNAs. In particular, vault RNA1-1 (vtRNA1-1) has emerged as a molecule of growing interest in the context of hematological malignancies. While its extracellular presence has been observed both freely in circulation and within exosomes, it is the latter that positions vtRNA1-1 within the framework of intercellular communication, particularly in the tumor microenvironment. Notably, vtRNA1-1 has been implicated in a range of oncogenic processes (apoptosis suppression; chemoresistance; autophagy regulation) primarily through its interactions with key molecular regulators (such as PSF and p62) and its modulation of survival pathways like PI3K/Akt and MAPK/ERK. Higher levels of vtRNA1-1 have been observed in patients with aggressive leukemia or bulky lymphoma, while a decline in vtRNA1-1 levels occurs during intensive chemotherapy [54]. Exosomal microRNAs (miRNAs) are also emerging as promising biomarkers for monitoring the efficacy of therapies in hematological malignancies. Recent studies have demonstrated that specific circulating miRNAs can be used as biomarkers to assess patient response to anticancer therapies. For example, miR-579-3p and miR-4488 have been identified as indicators of treatment response in melanoma, suggesting that similar approaches may also be applicable to hematological malignancies [55]. By categorizing the prominent hematological malignancies, the following section aims at examining how specific exosomal constituents, particularly lncRNAs and miRNAs, function as potential biomarkers across the spectrum of blood neoplastic disorders.

### 4.1. Hematological Malignancies of Lymphoid Lineage

#### 4.1.1. Exosomal miRNAs and Multiple Myeloma (MM)

Multiple Myeloma (MM) is the second most common hematologic malignancy worldwide, with an estimated annual incidence of 7.1 per 100,000 men and women. The main feature of the disease is B-cell and plasma-cell (PC) proliferation in the bone marrow (BM) and in extramedullary (EM) organs, with the secretion of monoclonal immunoglobulins (Igs) described as monoclonal (M) protein. In 10–20% of cases, patients are asymptomatic and present with ≥10% PCs in the BM, a condition described as smoldering myeloma (SMM), which does not require any treatment. On the other hand, MM is described as an active disease when a patient develops several pathological conditions and organ damage such as hypercalcemia, lytic bone lesions, anemia, renal insufficiency, and hyper-viscosity. This severe clinical picture needs immediate treatment and supportive care [56]. One of the most aggressive forms is represented by extramedullary (EMD) localization which portends poor prognosis [57] and, despite great advances in therapeutic strategies and various real-life experiences [58], there is still absence of a specific therapeutic consensus [56]. Di Noto et al. isolated and analyzed exosomes from MM, monoclonal gammopathies of undetermined significance (MGUS), and healthy individuals. The study found that MM patients produce approximately four times more exosomes than those with MGUS or healthy controls [59]. Emerging evidence suggests that exosomal miRNAs are instrumental in the pathogenesis of MM and related monoclonal gammopathies and are considered promising biomarkers for detecting disease progression [60]. One study revealed that the levels of specific exosome-derived miRNAs, namely *miRNA-20a-5p*, *miRNA-103a-3p*, and *miRNA-4505*, were significantly different among patients with MM, smoldering multiple myeloma (SMM), and healthy individuals. This suggests their potential utility in differentiating stages or types of disease and, more specifically, their useful role as early indicators of disease transformation from indolent monoclonal gammopathies to overt MM [61].

Specifically, *miR-20a-5p* presents a double function. On one hand, it is able to interact with various targets including runt-related transcription factor 3 (*RUNX3*) [62], *Rab27B* and *Smad 4*, inducing proliferation, invasion, metastasis and radio-resistance [63]. On the other hand, it exerts negative regulatory effects on autophagy-related gene 7 (*ATG7*), causing apoptosis and repressing proliferation [64]; *miR-20a-5p* also leads to chemoresistance by modulating *MAPK/ERK* and *cAMP/PKA* functions [65]. *miR-103a-3p* promotes tumor cell proliferation and invasion by targeting and silencing the phosphatase and tensin homolog (*PTEN*) gene (a tumor suppressor gene) and activating the *PI3k-Akt* pathway (which is generally downregulated by *PTEN*) [66]. Pula et al. have recently included *miR-103a-3p* among those miRNAs involved in resistance to Bortezomib, a proteosome inhibitor employed in standard regimens to treat MM [67]. Furthermore, *miR-103a-3p* can suppress phosphorylation of Yes-associated protein (YAP), a key effector of the Hippo pathway, thereby enhancing cell proliferation and tumor progression [68]. The third miRNAs, *miR-4505*, though less well characterized, has been linked to transcriptional regulation of heat shock proteins (HSPs), which can support proteostasis and survival in malignant plasma cells [61]. Interestingly, while *miR-20a-5p* and *miR-103a-3p* are generally regarded as tumor-promoting, their exosomal levels were found to decrease progressively from HCs to SMM to MM, suggesting a complex regulation of secretion versus intracellular retention. Conversely, *miR-4505* levels increased with disease progression, possibly reflecting an adaptive mechanism to enhance stress response pathways in advanced disease [61].

In a separate analysis, researchers identified several differentially expressed miRNAs (DEMs) originating from bone marrow stromal cells (BMSCs) that could distinguish MM patients from both healthy individuals and those with MGUS. Specifically, data showed distinct expression patterns of *hsa-miRNA-10a* (increased) and *hsa-miRNA-16* (decreased). These miRNAs regulate key genes such as *IGF1R* and *CCND1*, which play crucial roles in MM development. Additionally, targets like *EPHA8*, *CUL3*, and *ELAVL1* may also be influenced by these miRNAs, indicating their relevance in disease progression and their potential as diagnostic or therapeutic targets [69]. In more detail, *miR-10a* is significantly upregulated and functions as an oncogenic miRNA, in part, by targeting *EPHA8*, a receptor involved in ephrin signaling and axon guidance. The downregulation of *EPHA8* by *miR-10a* has been linked to increased expression of *SEMA5A*, a guidance molecule whose overexpression correlates with enhanced tumor proliferation, metastasis, and poor patient outcomes. In contrast, *miR-16*, which functions as a tumor suppressor, is markedly downregulated in MM, leading to the upregulation of several oncogenic targets. These include *IGF1R* and *CCND1*, both of which contribute to MM cell growth and survival through activation of the *PI3K-Akt* signaling pathway and promotion of cell cycle progression, respectively. Additionally, the reduction in *miR-16* results in the upregulation of *CUL3*, an *E3* ubiquitin ligase that has been implicated in stabilizing cyclin *D1*, thereby enhancing its oncogenic activity. Another relevant target is *ELAVL1*, an RNA-binding protein known to stabilize transcripts of pro-proliferative genes such as *FUT4*, *IGF1R*, and *CCND1*. Elevated levels of *ELAVL1*, facilitated by *miR-16* downregulation, may further amplify growth-promoting signals in MM cells. Collectively, these data suggest that the exosomal *miR-10a-EPHA8-SEMA5A* and *miR-16-IGF1R/CCND1-CUL3/ELAVL1* regulatory axes represent crucial mechanistic pathways underlying MM progression [69].

In addition to miRNAs, long non-coding RNAs (lncRNAs) encapsulated in exosomes have also demonstrated diagnostic relevance in MM. One such molecule, *PRINS* (Psoriasis Susceptibility-Related RNA Gene Induced by Stress), was found to be differentially expressed in serum exosomes from MM and MGUS patients compared to healthy individuals. Quantitative analysis showed that exosomal *PRINS* levels distinguished MM and MGUS subjects from healthy controls with a specificity of 83.3% and sensitivity of 84.9%, indicating high diagnostic potential. Moreover, *PRINS* expression was closely associated with well-established chromosomal aberrations observed in MM, including deletion 13q14 (del(13)(q14)), deletion 17p13 (del(17)(p13)), translocation t(4;14), gain of 1q21 (gain(1)(q21)), and hyper-diploidy. These correlations suggest that exosomal *PRINS* may reflect underlying genomic instability and serve as a biomarker for risk stratification and disease progression in patients with monoclonal gammopathies [70]. Furthermore, it has been studied that high-risk multiple myeloma (HR-MM) cells secrete a great number of hypoxic exosomes containing *miR-135b,* which targets and inhibits the *FIH-1* [the factor inhibiting the hypoxia-inducible factor (HIF-1α)] in ECs of the bone marrow [71]. In this way, MM cells create a hypoxic environment which stimulates pro-tumoral neo-angiogenesis. Lastly, it should be mentioned that despite numerous therapeutic lines, it is still not possible to eradicate the disease and MM inexorably relapses. The onset of multidrug resistance provokes the occurrence of a refractory disease. A continuous and bidirectional exchange of information takes place between the microenvironment and neoplastic cells to solicit the demands of cancer cells, and miRNAs are involved in promoting drug resistance [72]. For example, *miR-21* mediates resistance to dexamethasone by downregulating *RhoB*, a key apoptotic regulator. Interestingly, *miR-21* is upregulated upon adhesion to bone marrow stromal cells (BMSCs), highlighting the role of the tumor microenvironment [72,73]. Chemoresistance appears to arise not from isolated miRNA alterations but from the convergent effect of miRNA expression profiles, which regulates multiple redundant survival pathways. Specific serum miRNA signatures, including elevated *miR-16-2-3p* [74], *miR-19b-3p*, and *miR-29b-3p*, or reduced *miR-30c-5p*, *miR-181a-5p*, and *miR-744-5p*, correlate with Bortezomib refractoriness. These miRNAs orchestrate transcriptional repression or activation of genes involved in cell cycle control (*CDK5*, *Snail1*), epigenetic regulation (*EZH2*, *PHF19*), and apoptotic signaling (*P53*, *BAX/BAK*), thereby conferring resistance [72]. Overall, miRNAs exert chemoresistance in MM by targeting key regulatory nodes across multiple oncogenic pathways, highlighting their potential as biomarkers and therapeutic targets to overcome drug resistance.

Lastly, Zhang et al. considered data from 204 patients affected by MM and found that bortezomib-tolerant patients are characterized by the downregulation of several specific exosomes, namely *miR-165p*, *miR-15a5p*, *vmiR-20a5p,* and *miR-175p* [75]. Lastly, Zhang et al. considered data from 204 patients affected by MM and found that bortezomib-tolerant patients are characterized by the downregulation of several specific exosomes, namely *miR-16-5p*, *miR-15a5p*, *vmiR-20a5p,* and *miR-17-5p* [75]. These differentially expressed miRNA families play key roles in post-transcriptional regulation by influencing transcription co-factors, the MAP kinase pathway and ubiquitin conjugating enzyme activity. Furthermore, it was found that these four miRNAs exhibited higher synergistic effects. This suggested a functional complexity where a global central core of the post-transcriptional regulatory network is involved as a Bortezomib-resistant mechanism of MM [75]. Interestingly, a study carried out in 2020, authors developed a synthetic oligo-single-stranded DNA that mimicked the sequence of human *miR-15a-5p*. To improve its stability and binding affinity, they chemically modified it using locked nucleic acids (LNA), creating *LNA-15a*. Scientists showed that *LNA-15a* potently suppressed cell growth and induced apoptosis in MM and other cancer cell lines in vitro. In particular, it targeted and downregulated key target genes, namely BCL-2, VEGF-A, and PHF19 (epigenetic modulator). It also enhanced the anti-MM effect of bortezomib in a synergistic manner in OCI-My5 MM cells. On the other hand, analyzing in vivo mouse models, scientists found that *LNA-15a* significantly inhibited tumor growth and prolonged survival compared with controls. Therefore, the study provides strong preclinical evidence that *LNA-modified miR-15a mimics* could serve as a promising therapeutic strategy for MM [76].

#### 4.1.2. Exosomal miRNAs and Chronic Lymphoid Leukemia (CLL)

Chronic lymphocytic leukemia (CLL) is the most frequent leukemia in the Western world. It is caused by the uncontrolled proliferation of clonal B-cells, featuring a peculiar immunophenotype expressing CD5, CD19, CD20(dim), and CD23. It mainly represents an indolent disease with a wide variety of treatment available, including targeted therapies such as Bruton tyrosine kinase (BTK) inhibitors (e.g., ibrutinib, acalabrutinib), *BCL-2* inhibitors (e.g., venetoclax), and monoclonal antibodies (e.g., rituximab, obinutuzumab), which are often used alone or in combination [77]. One of the most frequently observed chromosomal abnormalities in CLL is the deletion of chromosome region 13q14.3, which contains *miR-15a* and *miR-16*. Therefore, in the majority of CLL cells, these 2 miRNAs are either absent or markedly reduced in expression. More specifically, the downregulation of *miR-15a/16-1*, often seen in aggressive CLL subtypes, leads to unchecked *BCL2* expression and resistance to apoptosis [78]. Focusing on exosomal miRNA, Moussay et al. provided evidence that specific extracellular miRNAs could serve as sensitive biomarkers for CLL, noting that *miR-195* and *miR-20a* are present at significantly different levels in the plasma of CLL patients compared to both healthy individuals and patients with other hematologic cancers. Furthermore, several of these circulating miRNAs, especially *miR-195*, *miR-29a*, *miR-222,* and *miR-150*, showed marked differences in concentration between ZAP-70+ and ZAP-70- CLL cases [79]. *MiR-195*, known for its tumor-suppressive properties, is significantly downregulated in *ZAP-70+* CLL, which correlates with enhanced mitochondrial fitness and cell proliferation via targets such as *mitofusin-2* and *PRR11*. This downregulation may contribute to the aggressive phenotype observed in ZAP-70+ cases [80]; *miR-29a*, another tumor suppressor, is also reduced in *ZAP-70+* CLL, allowing overexpression of oncogenic targets like *TCL1* and anti-apoptotic proteins such as *MCL1*, thereby promoting survival and proliferation [81]. Conversely, *miR-222* appears to be upregulated in *ZAP-70+* CLL, where it targets *PTEN* and *p27Kip1*, facilitating activation of the *PI3K/AKT* pathway and reducing apoptosis, consistent with the enhanced survival signaling driven by *ZAP-70* [82]; *miR-150* is expressed at lower levels in *ZAP-70+* cases, leading to increased expression of *FOXP1* and *GAB1* [key enhancers of B-cell receptor (*BCR*) signaling]. This amplification of *BCR* signaling is associated with poorer prognosis and shorter treatment-free survival [83]. Collectively, these miRNAs modulate key signaling pathways, such as *AKT*, *MYC,* and *BCR*, that are central to the pathobiology of CLL and its clinical heterogeneity. In a multivariate model, higher *miR-20a* expression was independently associated with a longer time to treatment initiation, supporting its positive prognostic relevance [78]. Studies have demonstrated that EVs release by CLL cells is dynamically regulated by microenvironmental stimuli such as B-cell receptor (*BCR*) ligation, CD40/IL-4 signaling, and Toll-like receptor (TLR) activation, all of which converge on NF-κB activation. More precisely, CD40/IL-4 signaling, which mimics T-cell-mediated activation of B-cells, does not significantly increase the quantity of EVs but modifies their molecular composition. In particular, exosomes result in a higher *miR-363* cargo. When transferred to CD4^+^ T-cells, *miR-363* induces downregulation of CD69, an early T-cell activation marker and alters CD4+ migration and immunological synapse function [84]. In contrast, TLR activation, particularly through CpG oligonucleotides (TLR9 ligands), has been shown to significantly increase the quantity of EVs released by CLL cells via *MyD88–NF-κB* signaling. These EVs are enriched in mRNAs related to BCR signaling kinases (e.g., *LYN*, *SYK*). This crosstalk between TLR and BCR signaling may potentiate leukemic cell survival, inflammatory signaling, and immune evasion [84]. As mentioned, patients affected by CLL exhibit impaired T-cell-mediated immunity. Emerging evidence indicates that CLL progression promotes the expansion of MDSCs, which inhibits T-cell activation and fosters the development of regulatory T-cells (Tregs) through the transfer of exosomal *miR-155* [85]. Mechanistically, exosomal *miR-155* is internalized by circulating monocytes, where it downregulates the expression of suppressor of cytokine signaling 1 (*SOCS1*), a key negative regulator of *STAT1* signaling. This results in enhanced *STAT1* activation and increased expression of indoleamine 2,3-dioxygenase (IDO), an immunosuppressive enzyme critical for T-cell inhibition. In parallel, *miR-155* triggers a metabolic reprogramming of monocytes toward aerobic glycolysis (a hallmark of MDSCs differentiation), thereby promoting their suppressive phenotype. Experimental blockade of *miR-155* prevents IDO induction and glycolytic switching, confirming its central role in MDSC polarization. Thus, miR-155 widely contributes to CLL-mediated immune evasion [84]. Furthermore, the exosome-mediated delivery of microRNAs to monocytes appears to play a role in immune evasion by CLL, particularly through the upregulation of *PDL1* expression [86]. The exosome-mediated delivery of microRNAs to recipient cells plays a critical role in immune evasion and paracrine microenvironmental remodeling by CLL. For example, stromal cells are converted into cancer-associated fibroblast through *AKT*, *ERK*, and *CREB* pathway activation [84]. In this regard, *miR-202-3p* plays an interesting role. A study utilizing a CLL cell line demonstrated that CLL-derived exosomes are rich in small RNAs such as *miR-202-3p* [87]. While intracellular levels of *miR-202-3p* are relatively low in CLL cells, this microRNA is selectively and consistently packaged into exosomes and transferred to surrounding stromal cells, such as HS-5 (a stromal cell line with fibroblast morphology used in the study) [87]. Once internalized, *miR-202-3p* directly targets the Suppressor of Fused (*SUFU*) mRNA. *SUFU* inhibits the activation of GLI transcription factors (GLI1/GLI2), which are downstream effectors of Hedgehog signaling pathway. Therefore, the suppression of *SUFU* by exosomal *miR-202-3p* relieves this inhibitory checkpoint, leading to the activation of Hedgehog signaling in recipient stromal cells. As a consequence, these stromal cells are converted into cancer-associated fibroblasts (CAFs). Hedgehog pathway activation in the tumor microenvironment has been associated with enhanced stromal support for leukemic cells, increased cellular proliferation, and resistance to therapy, all of which contribute to CLL progression. Interestingly, *SUFU* expression is elevated in *IgVH*-unmutated CLL, a subgroup associated with worse prognosis, and negatively correlates with *miR-202-3p* levels [88]. Exosomes originating from CLL exhibit a refined enrichment of microRNAs, most notably those belonging to the *miR-150*, *miR-155*, and *miR-29* families, as well as *miR-223*, while displaying but a modest presence of transfer RNAs and short ribosomal RNAs [89]. This distinguished microRNA profile may, with due precision, serve to set CLL exosomes apart from those associated with other hematological disorders [90]. Additionally, altered miRNA expression profiles have been identified in CLL cells. These profiles are not merely passive reflections of disease state but actively modulate pathways central to leukemogenesis and progression. For instance, overexpression of *miR-155* and *miR-21*, frequently observed in *ZAP-70*-positive and *IgVH*-unmutated cases, promotes cell survival by targeting tumor suppressors such as *SHIP1*, *PTEN*, and *PDCD4*, thereby enhancing *AKT* and *STAT3* signaling. Collectively, these miRNA alterations contribute to CLL pathogenesis and provide support for their use as diagnostic and predictive tools [78].

#### 4.1.3. Exosomal miRNAs and Lymphomas

Lymphomas represent a broad and biologically diverse group of hematological malignancies that originate from the clonal proliferation of lymphocytes. This category encompasses numerous subtypes, the most prominent being Hodgkin lymphoma (HL) and non-Hodgkin lymphomas (NHLs). Several studies have found that patients with Hodgkin lymphoma display significantly elevated levels of EV-associated miRNAs, including *miR-24-3p*, *miR-127-3p*, *miR-21-5p*, *miR-155-5p*, and *Let-7a-5p*, in their plasma compared to healthy individuals. In classical HL patients, these miRNAs were highly expressed prior to treatment initiation. Importantly, their levels were observed to decline in patients who achieved a complete metabolic response to therapy. However, a rebound in miRNA expression occurred in patients who later relapsed, indicating their potential utility in disease monitoring and early relapse detection [91]. Among these, *miR-21-5p* and *miR-155-5p* act as potent oncomiRs. The former downregulates *PTEN* and programmed cell death protein 4 (*PDCD4*), leading to enhanced *PI3K/AKT* and *NF-κB* signaling, which promotes tumor cell survival and immune evasion [92]. Similarly, *miR-155-5p* targets suppressors of cytokine signaling 1 (*SOCS1*) and Src homology 2 domains containing inositol 5-phosphatase 1 (*SHIP1*), de-repressing *JAK/STAT* signaling, and contributing to lymphomagenesis [93]. In the case of Diffuse Large B-Cell Lymphoma (DLBCL), the most common subtype of NHL, exosomes and their RNA cargo have shown significant promise as non-invasive biomarkers. One study identified five miRNAs, namely *hsa-miR-379-5p*, *hsa-miR-135a-3p*, *hsa-miR-4476*, *hsa-miR-483-3p*, and *hsa-miR-451a*, that are present at altered levels in DLBCL patients’ plasma-derived exosomes. Among these, *miR-379-5p*, *miR-135a-3p*, and *miR-4476* were upregulated in patients, whereas *miR-483-3p* and *miR-451a* were found to be downregulated compared to healthy controls. These distinct expression profiles highlight the diagnostic potential of exosomal miRNAs in distinguishing DLBCL patients from unaffected individuals [94]. The pro-tumoral role of *miR-4476* has been analyzed in glioma cells. According to the study by Lin et al., *miR-4476* directly targets adenomatous polyposis coli (APC), a negative regulator of the *Wnt/β-catenin* signaling pathway. This suppression of APC leads to activation of *β-catenin*, which in turn upregulates *c-Jun*, a transcription factor that enhances *miR-4476* expression by binding upstream of its transcription start site. This loop amplifies oncogenic signaling, resulting in increased cell proliferation, migration, and invasion of neoplastic cells [95]. Moreover, certain exosomal miRNAs have been correlated with clinical outcomes in DLBCL. For instance, high levels of *miR-125b-5p* and *miR-99a-5p* have been associated with shorter progression-free survival (PFS). Conversely, reduced levels of *miR-107* and *miR-451a* have been linked to poor prognosis [17,96]. Mechanistically, *miR-125b-5p* contributes to poor prognosis by targeting *BCL-2*, *LIN28B*, and *TP53*, leading to impaired apoptosis and unchecked tumor growth [97]. Concurrently, *miR-99a-5p* regulates the *mTOR*, *IGF-1R*, *FGFR3, SMARCA5*, and *SMARC1* axis, facilitating cell proliferation, therapy resistance, and epigenetic reprogramming [98]. By contrast, *miR-451a*, typically downregulated in high-risk patients, normally suppresses macrophage migration inhibitory factor (*MIF*) modulating metabolic homeostasis and oxidative stress through AMP-activated protein kinase (*AMPK*) signaling, thus inducing cell cycle arrest and apoptosis when expressed [99]. Furthermore, comparing parental DLBCL cells to those cells resistant to R-CHOP therapy, it was found that *miR-99a-5p* and *miR-125b-5p* were consistently dysregulated [100]. This suggests their potential as biomarkers of chemoresistance [17]. Focusing on the prognostic role of exosomal *miR-451a*, when it is combined with the International Prognostic Index (IPI), its expression may enhance predictive accuracy for both PFS and overall survival (OS) in DLBCL patients [101]. On the other hand, *miR-155* has emerged as a highly informative biomarker not only in DLBCL but also in monoclonal B-cell lymphocytosis and CLL. In patients with refractory or relapsed DLBCL, exosomal *miR-155* levels were found to be significantly higher compared to those still undergoing R-CHOP or who had responded well to treatment [102]. A comprehensive profiling study examining the entire plasma mirnome in DLBCL, including exosomal components, revealed several notable alterations. Elevated levels of *miR-124* and *miR-532-5p* were observed in patient plasma, while *miR-425*, *miR-141*, *miR-145*, *miR-197*, *miR-345*, *miR-424*, *miR-128*, and *miR-122* were all decreased. Importantly, the increased expression of *miR-20a*, *miR-20b*, *miR-93*, *miR-106a*, and *miR-106b* was associated with higher mortality rates, suggesting a significant prognostic role for these miRNAs [103]. In particular, these fve miRNAs promote malignancy by suppressing *E2F1*, *p21*, and components of the *TGF-β* signaling cascade, thereby enhancing proliferation and limiting apoptosis [104]. Additionally, recent findings show that these miRNAs can downregulate dual specificity phosphatase 2 (*DUSP2*), further contributing to oncogenic signaling in lymphoma models. This phosphatase belongs to the MAP kinase phosphatase family and specifically dephosphorylates threonine and tyrosine residues on MAPKs, including ERK1/2, p38, and JNK, thereby attenuating MAPK oncogenic signaling cascades [105]. Notably, normal B-cells that internalized exosomes from DLBCL patients showed subtype-specific miRNA expression changes, suggesting that exosomes can reprogram recipient cells based on the genetic characteristics of their origin. Among the altered miRNAs, *miR-3960*, *miR-6089*, and *miR-939-5p* were proposed as a diagnostic signature for DLBCL [106]. In detail, miR-3960 targets genes such as HOXA2 and RUNX2, which may influence stromal remodeling and extracellular matrix interactions in the lymphoma niche; miR-6089 suppresses TLR4-mediated NF-κB signaling by directly targeting TLR4 mRNA, thus attenuating pro-inflammatory cytokine release facilitating immune evasion. miR-939-5p has been reported to downregulate VEGFA and NOS2, key mediators of angiogenesis and nitric oxide signaling, which can reshape the metabolic and vascular characteristics of the tumor microenvironment [106]. This data support the hypothesis that exosomes not only serve as disease markers but also play an active role in modifying the molecular phenotype of neighboring or distant cells, thereby contributing to lymphoma heterogeneity and progression.

#### 4.1.4. Exosomal miRNAs and Acute Lymphoblastic Leukemia (ALL)

Acute lymphoblastic leukemia (ALL) is a malignant disorder of the bone marrow characterized by the uncontrolled proliferation and accumulation of immature lymphoid cells, known as lymphoblasts, which originate from early precursors of B or T lymphocytes and replace normal hematopoietic elements in the BM. ALL is the most common cancer in children but can also affect adults, where it typically follows a more aggressive clinical course [107]. A key genetic abnormality is the presence of the Philadelphia (Ph) chromosome, which arises from a reciprocal translocation between the long arms of chromosomes 9 and 22, designated t(9;22)(q34;q11). This translocation can be found in 30% of ALL cases [108] and implies the fusion of the *BCR* (breakpoint cluster region) gene on chromosome 22 with the *ABL1* (Abelson murine leukemia viral oncogene homolog 1) gene on chromosome 9, resulting in the formation of the BCR-ABL1 fusion gene. The *BCR-ABL1* protein is a constitutively active tyrosine kinase that drives leukemic cell proliferation and survival, and its presence is associated with a poorer prognosis unless specifically targeted with tyrosine kinase inhibitors (TKIs), which have become a cornerstone of treatment in Ph-positive ALL [107]. Studies have shown that exosomes derived from Precursor B-cell ALL (pre-B ALL) cells, particularly in patients who are Philadelphia chromosome-positive (Ph+), have the capacity to stimulate the proliferation of otherwise quiescent pre-B ALL cells. These exosomes are rich in specific miRNAs which interact dynamically with the surrounding cellular environment. These miRNAs may target key tumor suppressors such as *PTEN* or *p21*, thereby relieving inhibition on proliferative signals such as *PI3K/AKT* and *CDK-cyclin* complexes [109]. Through this interaction, they promote the expansion of B-ALL cells by enhancing cellular proliferation and migration, likely via activation of pathways such as *MAPK/ERK* and downregulation of adhesion-regulating genes, while concurrently modifying the tumor microenvironment to suppress immune surveillance mechanisms through the induction of immune checkpoint molecules like *PD-L1* or the downregulation of MHC class II expression in antigen-presenting cells [110,111]. In a recent investigation, researchers explored the proteomic composition of plasma-derived exosomes in this category of patients. The study identified 342 differentially expressed proteins (DEPs) between patients and controls, indicating significant molecular alterations within the exosomal content. Among the upregulated proteins were *ADAM17* and *ATG3*, both of which may contribute to disease pathogenesis. Specifically, the former is believed to play a role in the activation of the *NOTCH* signaling pathway by cleaving *NOTCH* ligands or receptors, leading to *NOTCH* intracellular domain (NICD) translocation to the nucleus and transcriptional activation of oncogenes like *MYC* and *HES1*, which is frequently implicated in leukemogenesis. The latter appears to be involved in the induction of autophagy, facilitating the formation of autophagosomes and maintaining leukemia cell viability under chemotherapeutic or metabolic stress by preventing apoptosis and supporting nutrient recycling [112]. These proteomic findings present exciting prospects for the development of novel diagnostic tools and therapeutic interventions for B-ALL, emphasizing the diagnostic and biological significance of this exosomal cargo. One of the most critical complications in ALL is Central Nervous System (CNS) relapse, which significantly worsens patient prognosis and increases the overall mortality rate. Although data on the involvement of exosomes and miRNAs in CNS infiltration are limited, findings by Hua Zhang et al. have shed light on this issue. Their study demonstrated that *miR-181a*, found in specific EV subtypes in the cerebrospinal fluid (CSF), serves as a highly sensitive biomarker for CNS involvement in ALL. The sensitivity of *miR-181a* detection in the CSF for identifying early CNS leukemia reached 90%, markedly outperforming traditional cytology methods, which had a sensitivity of only 54.5% [113,114]. Furthermore, *miR-181a* has been shown to selectively induce B-lymphocyte proliferation, particularly in pediatric ALL (P-ALL) by downregulating genes such as *EGR1*, which functions as inhibitors of the cell cycle [115,116]. Researchers have also successfully incorporated *miR-181a* inhibitors into exosomes, demonstrating that these engineered exosomes can suppress proliferation induced by wild-type exosomes. The inhibition mechanism involves downregulation of key pro-survival genes (*MCL-1*, *BCL2*) and proliferative genes (*PCNA*, *Ki-67*), thereby promoting intrinsic apoptotic signaling and cell cycle arrest at the G1/S checkpoint [117]. This innovative approach offers the possibility to deliver therapeutic RNA molecules through exosomes. Additionally, another study has demonstrated that *miR-181b-5p* is capable of modulating cell cycle and suppressing apoptosis in ALL cells. At the molecular level, *miR-181b-5p* may target pro-apoptotic genes such as *BIM* (a pro-apoptotic member of the BCL2 family) or regulators of cell cycle checkpoints like *CDC25A* or Synovial Sarcoma X breakpoint 2 Interacting Protein (SSX2IP), allowing leukemic cells to evade death signals and continue proliferating [118]. From a mechanistic perspective, *CDC25A* promotes the G1/S and G2/M transitions by activating the *CDK2/cyclin E* and *CDK1/cyclin B* complexes. The suppression of *CDC25A* mediated by *miR-181b-5p* paradoxically stabilizes uncontrolled proliferation, as it disrupts checkpoint control; SSX2IP is protein involved in spindle assembly, centrosome maturation, and chromosome segregation during mitosis, thus safeguarding genomic integrity. Downregulation of SSX2IP by *miR-181b-5p* promotes aneuploidy and supports leukemic proliferation [119].

All key exosomal microRNAs listed in hematological malignancies of the lymphoid lineage described in the upper sections are summed up in Table 1.

### 4.2. Hematological Malignancies of Myeloid Lineage

#### 4.2.1. Exosomal miRNAs and Chronic Myeloid Leukemia

Chronic myeloid leukemia (CML) is a myeloproliferative disorder driven by the formation of the *BCR-ABL* fusion gene, which arises from a reciprocal translocation between chromosomes 9 and 22, commonly referred to as the Philadelphia chromosome. Recent studies analyzing extracellular vesicles isolated from two different CML cell lines discovered a distinct RNA band of approximately 250 base pairs (bp). Sequencing of this RNA fragment revealed an astounding 99% sequence homology with a portion of mRNA encoding the *BCR-ABL* chimeric protein, thereby demonstrating exosomes released by CML cells harbor tumor-specific transcripts [120]. Remarkably, this nearly 250 bp band was also detected in exosomes isolated from the plasma of CML patients, particularly those in the blast crisis or accelerated phases of the disease. These findings present strong evidence that CML-derived exosomes can serve as reliable vehicles for identifying and detecting the *BCR-ABL* fusion transcript, offering an innovative, non-invasive platform for molecular diagnostics in CML [121]. Exosomes derived from K562 cells, a cell line representative of the erythroleukemia subtype of CML, have been demonstrated to induce the phosphorylation of Src kinase, a known downstream target of dasatinib, a TKI used in CML treatment. The activation of Src subsequently triggers a cascade of downstream signaling events in endothelial cells (ECs), suggesting that CML-derived exosomes can influence the surrounding stromal and vascular environment [122]. Further studies revealed that *miR-92a-1-5p*, a microRNA encapsulated within K562-derived exosomes, targets and downregulates integrin α5 expression in ECs. This downregulation enhances ECs migration and promotes the formation of tubular-like structures. This represents a crucial step in neo-angiogenesis and the establishment of a pro-leukemic vascular niche [123,124]. Importantly, *miR-92a-1-5p* is a member of the oncogenic *miR-17~92* cluster (*miR-17*, *miR-18a*, *miR-19a*, *miR-20a*, *miR-19b-1,* and *miR-92a-1-5p*), which plays a central role in CML leukemogenesis [125]. This cluster regulates essential cellular processes such as proliferation, apoptosis, and autophagy. Expression of *miR-92a-1-5p* is regulated by BCR::ABL1 and is consistently downregulated by tyrosine kinase inhibitors (TKIs) like imatinib. Functional studies revealed that *miR-92a-1-5p* inhibition reduces proliferation and enhances apoptosis, while its overexpression promotes leukemic cell survival and partially counteracts imatinib-induced apoptosis. These effects are mediated through direct targeting of *TP53INP1*, a tumor suppressor involved in cell cycle arrest, and *BNIP3L*, a regulator of autophagy and apoptosis. Inhibition of *TP53INP1* reverses the anti-proliferative effect of *miR-92a-1-5p* suppression, while *BNIP3L* inhibition counteracts the increase in autophagosomes induced by *miR-92a-1-5p* inhibition. Together, these findings suggest that miR-92a-1-5p is a promising therapeutic target in CML [126].

Additionally, another exosomal miRNA, *miR-210*, was found to interact with *EPHRIN-A3*, a gene involved in both angiogenesis and VEGF-mediated signaling pathways [127]. Lastly, exosomal transfer of *miR-126* to ECs directly targeted the 3′UTR of *CXCL 12* (which guides chemotaxis via *CXCR4*) and *VCAM1* (which mediates cell adhesion to endothelial/stromal cells) mRNA [128], thus modulating adhesive and migratory abilities of CML cells. All these interactions underscore the mechanistic involvement of specific exosomal miRNA-mediated pathways in facilitating pro-leukemic vascular remodeling processes and promoting tumor expansion and progression. One of the most common causes of resistance in CML are point mutations in the ABL kinase domain. These mutations severely impair drug binding and compromise treatment efficacy. Recent studies have highlighted the complex and context-dependent role of *miR-221* in various cancers, including CML. Data showed that *miR-221* expression is significantly downregulated in CML patients with poor response to imatinib, and similarly in resistant *K562/G* cells. This suggests that epigenetic silencing of *miR-221* may contribute to drug resistance [129]. Functionally, overexpression of *miR-221* in *K562/G* cells inhibited proliferation and enhanced apoptosis, thereby increasing sensitivity to imatinib. Protein profiling revealed that *miR-221* altered the expression of key apoptosis-related proteins, including BCL-2, Bax, Caspase-3, survivin, and p27. Notably, suppression of survivin, a known anti-apoptotic protein, has been shown to sensitize resistant CML cells to TKIs [130]. Signal Transducer and Activator of Transcription 5 (STAT5) is a family of transcription factors that play a central role in how cells respond to external signals (like cytokines and growth factors). There are two closely related forms: STAT5A and STAT5B [131]. This factor is frequently overexpressed and hyperactivated in TKI-resistant CML cells, and its inhibition has been linked to restored drug sensitivity [132]. In more detail, data showed that overexpression of *miR-221* reduced STAT5 and p-STAT5 levels, while its inhibition elevated them. Therefore, these findings identify STAT5A and STAT5B as *miR-221* targets and demonstrate that downregulation of STAT5B promotes apoptosis and reduces chemoresistance [129]. Moreover, STAT5 activity has been associated with increased reactive oxygen species (ROS) and DNA damage, which can drive secondary mutations and further resistance [133]. In conclusion, targeting the STAT5 signaling pathway may offer a novel therapeutic strategy, particularly for TKI-resistant patients. However, the full molecular landscape of *miR-221* in CML remains to be elucidated and should be explored in future research.

#### 4.2.2. Exosomal miRNAs and Myelodisplastic Syndromes

These clonal hematopoietic neoplasms are defined by cytopenias and morphologic dysplasia. According to the 5th edition of the World Health Organization (WHO) Classification (2022), the recommended threshold for dysplasia is set at 10% for all cellular lineages. Moreover, Myelodisplastic syndromes (MDS) entities are now grouped as those having defined genetic abnormalities and those that are morphologically defined. If no defining genetic aberrations are detected, an arbitrary cut off of 20% blasts to distinguish MDS from AML is retained [134].

Previous research underscored the significant potential of EVs-derived miRNAs as non-invasive diagnostic biomarkers in MDS. Giudice et al. conducted a comprehensive screening of plasma exosomal miRNAs in MDS patients at diagnosis, identifying 25 miRNAs uniquely expressed in patients with MDS and/or aplastic anemia (AA) [135]. Among these, 14 miRNAs were specific to MDS, while *miR-196a-5p*, *miR-196b-5p*, *miR-4267*, and *miR-378i* were common to both MDS and AA, highlighting their potential to discriminate between these clinically overlapping disorders [135]. These miRNAs are implicated in the regulation of critical pathways governing hematopoietic stem cell (HSC) differentiation and survival, including *HOXA* (*miR-196a/b*), *ERK5*, *PTEN* (*miR-378i*) [136], *STAT3* (*miR-4267*), and VEGF signaling cascades [135]. Notably, elevated *miR-126-5p* levels correlated with poor responses to immunosuppressive therapy and reduced progression-free survival in AA patients, emphasizing its prognostic value [135]. At a molecular level, *miR-126 5p* promotes HSCs quiescence and resistance to apoptosis by targeting the long non-coding RNA Pituitary Tumor-Transforming Gene 3 (*PTTG3P*). In MDS, overexpression of *PTTG3P* induces apoptosis and drives HSPCs out of G_0_/quiescence into the cell cycle. Therefore, *PTTG3P* functions as a tumor suppressor: its upregulation sensitizes malignant progenitors to hypomethylating agents or decitabine. Conversely, *miR-126*, which is elevated in MDS stem/progenitor cells, directly binds to and suppresses *PTTG3P* transcript. As a consequence, this favors malignant stem cell self-renewal and results in a lower OS [137]. Several EV-derived miRNAs, such as *miR-125a*, *let-7a*, *miR-194-5p*, miR-22, *miR-661*, *miR-181a*, *miR-210*, and *miR-196-5b*, have been associated with adverse prognosis and disease progression. For example, *miR-125a* suppresses *TP53* and pro-apoptotic *BAX*, promoting survival [138], *let-7a* is associated with intermediate/high-risk karyotype MDS and targets *RAS/MYC*, thereby influencing proliferation [139], *miR-194-5p* targets *DNMT3A*, affecting epigenetic stability, *miR-181a* is elevated in MDS patients progressing to AML [140] and both *miR-181a* and *miR-210* modulate hypoxia-inducible and apoptotic pathways [138], and *miR-196-5b* (which is upregulated in high-risk MDS and is associated with transformation to AML) can target cell cycle inhibitors such as *CDKN1B* to drive aberrant proliferation [138]. In addition, repression of miRNAs targeting *DNMT1* (e.g., *miR-148a*, *miR-29b*) correlates with azacitidine resistance [138]. An interesting in vitro experiment has been carried out analyzing the role of *miR-661* in MDS. Data from the study showed that transfection with a *miR-661* mimic induced apoptosis in a myeloid cell’s lineage, primarily through activation of the *p53* pathway, which is known to regulate several pro-apoptotic miRNAs [141]. These findings align with previous research in other cancers (i.e., glioma, breast cancer, and osteosarcoma) where *miR-661* overexpression suppressed proliferation and promoted apoptosis. However, the relationship between *miR-661* expression and specific MDS subgroups remains unclear. Earlier studies reported variable expression across MDS subtypes but could not find a statistically significant association, possibly due to differences in detection methods and limited sample size. Similarly, although patients with high *miR-661* expression tended to fall into poorer prognostic categories [according to the Revised International Prognostic Scoring System (IPSS-R)], the trend lacked statistical significance. Overall, this study was the first to demonstrate that *miR-661* upregulation may contribute to the hallmark apoptosis seen in MDS, potentially via *p53*-mediated pathways [141].

Conversely, in del(5q) MDS patients undergoing lenalidomide therapy, elevated exosomal *miR-145* levels are linked to favorable clinical responses and transfusion independence (TI) [138]. This is due to the fact that *miR-145* alleviates ineffective erythropoiesis and promotes erythroid maturation by directly suppressing the ribosomal protein *RPS14* and components of the *TLR4/NF*-*κB* axis [142]. Mongiorgi et al. explored the interplay between *miR-192-5p* and *BCL2* gene expression within an experimental model. Their findings demonstrated that *miR-192-5p* directly targets the *BCL2* promoter, with reduced *BCL2* expression observed in MDS patients who respond to therapy, suggesting a suppressive role for *miR-192-5p* that may inhibit cellular proliferation in this subgroup. Building on these insights, prognostic evaluation of *miR-192-5p* within an MDS patient cohort revealed that elevated *miR-192-5p* levels at the fourth cycle of azacitidine plus lenalidomide (AZA + LEN) therapy significantly correlate with improved OS and leukemia-free survival (LFS). Consequently, *miR-192-5p* may serve as a valuable biomarker for stratifying patients into responders, those losing response, and non-responders, thus refining therapeutic decision-making [143].

#### 4.2.3. Exosomal miRNAs and Acute Myeloid Leukemia

Acute myeloid leukemia (AML) is a hematologic malignancy characterized by the abnormal proliferation and impaired differentiation of myeloid progenitor cells. This dysregulation leads to an accumulation of immature cells in BM and peripheral blood (PB), which disrupts normal hematopoiesis [144]. Recent scientific investigations have increasingly emphasized the diagnostic and prognostic potential of exosomes in AML. More specifically, AML exosomes present RNAs whose transcripts are implicated in AML pathogenesis, prognosis (*NPM1*, *FLT3-ITD*), response to therapy (*CXCR4, IGFIR*), and leukemic niche formation (*IGF-IR*, *CXCR4*, *MMP9*) [145]. Moreover, leukemia-derived TEXs are characterized by a 5-to-13-fold enrichment in parental miRNAs [146]. The stable encapsulation of lncRNAs within exosomes offers a promising avenue for their use as non-invasive biomarkers in diagnosis, prediction, and disease monitoring [147].

Among IncRNAs that can serve as valuable AML biomarkers, there are *LINC00265*, *LINC00467*, *UCA1,* and *SNHG1,* which are significantly dysregulated compared to healthy donors. In particular, *LINC00265*, *LINC00467,* and *UCA1* are downregulated, whereas *SNHG1* is upregulated. Furthermore, when used in combination, this exosome panel may offer a robust approach to enhance diagnostic accuracy [148]. *LINC00265* functions as an oncogenic lncRNA by activating the PI3K-AKT signaling pathway, thereby promoting cell proliferation and survival, and its high expression correlates with poor prognosis in AML patients [149]. Additionally, *LINC00265* regulates autophagy and apoptosis through the *miR-485-5p/IRF2* axis, where it sequesters *miR-485-5p* in order to upregulate *IRF2*, enhancing autophagy and suppressing apoptosis [150]. *LINC00467* facilitates AML progression by targeting the *miR-339/SKI* pathway; it suppresses *miR-339*, leading to increased expression of the oncogene *SKI* [151]. In pediatric AML, *UCA1* promotes cell proliferation and inhibits apoptosis by sequestering *miR-204*, which results in the upregulation of *SIRT1*, a survival-promoting factor. Silencing *UCA1* reverses these effects, limiting proliferation and accelerating apoptosis [152]. Upregulated *SNHG1* in AML sequesters the tumor-suppressive *miR-488-5p* in the cytoplasm of leukemic cells. Reducing *miR-488-5p* availability leads to upregulation of *NUP205*, a nuclear pore protein involved in cell cycle progression and chromatin regulation [153].

MicroRNAs encapsulated in AML plasma exosomes are linked to disease prognosis. For instance, elevated levels of exosomal *miR-532* have demonstrated a positive correlation with OS (although its exact mechanism remains unclear). However, no significant correlations were observed between *miR-532* expression and conventional clinical variables (including age, white blood cell count, French–American–British (FAB) classification subtypes, and cytogenetic risk categories) or the presence of mutations such as *FLT3-ITD*, *NPM1*, *CEBPA*, and *DNMT3A* [154]. On the other hand, high circulating levels of *miR-125b* are significantly associated with poor clinical outcomes, including a greater likelihood of relapsing and increased overall mortality [155]. Moreover, *miR-125b* was identified as an independent prognostic factor specifically for patients with intermediate-risk AML, emphasizing its potential utility in patient risk stratification [156]. In detail, *miR-125b* transported in AML-derived exosomes targets apoptotic regulators such as *BAK1* and *CBFβ*, suppressing *p53*-driven apoptosis and promoting leukemic blast survival and proliferation. It also fosters angiogenesis via upregulation of *VEGFA* and inhibits differentiation through *TET2* suppression [155]. Additional research has demonstrated that AML-derived exosomes are characterized by significantly elevated expression of *EV-miR-10b*, especially in those patients who present with normal cytogenetics. This increase was strongly correlated with more aggressive disease characteristics and shorter OS, solidifying the role of this miRNA as an independent prognostic biomarker. The high diagnostic value of *EV-miR-10b* showed a sensitivity of 82.50% and specificity of 77.89% [157]. Functionally, *miR-10b* can inhibit apoptosis and homeobox D10 expression in AML cells by directly targeting homeobox D10 [155]. Further research has identified a subset of serum-derived exosomal miRNAs, namely *miR-150*, *miR-155*, and *miR-1246* [158]. These miRNAs were proposed as potential early detection biomarkers due to their consistent presence and upregulation in patient samples, suggesting a role in AML pathophysiology. For example, *miR-155* blocks tumor suppressor *SHIP1* and *SOCS1*, enhancing *JAK/STAT* and *NF-κB* signaling, thereby promoting leukemogenesis and contributing to immune evasion [159]; *miR-1246* is internalized in leukemic stem cells (LSCs) where it targets/represses *LRIG1*, a negative regulator of *JAK/STAT* signaling. This results in the activation of *JAK2* and *STAT3*, promoting LSC survival and colony formation and blocking their differentiation [160].

Another significant finding involves *miR-425-5p*, which is a microRNA packaged within BM MSCs-derived exosomes. It has been hypothesized that this miRNA may play a functional role in modulating gene regulatory networks in AML. A comparative analysis has been conducted in order to study microRNA profiles from AML patient-derived MSCs and healthy controls. This miRNA was one of five candidate miRNAs found to be significantly downregulated in AML-derived exosomes. This finding did not reflect intrinsic changes in miRNA expression within the MSCs themselves, but rather a selective exosomal loading with specific miRNAs. Furthermore, *miR-425-5p* was predicted to directly inhibit a panel of 23 target genes. Conversely, its downregulation contributes to the upregulation of these predicted targets within the leukemic BM microenvironment. The panel included molecules involved in nuclear transport (e.g., *HNRNPA3*), immune modulation (e.g., *IFITM1*, *KIR3DS1*) and gene expression regulation (e.g., multiple zinc-finger proteins), implying that reduced *miR-425-5p* levels may facilitate an oncogenic shift in the stromal niche. Most of these targets showed increased expression in AML CD34^+^ cells compared to controls and this was consistent with reduced *miR-425-5p* function. However, only Apolipoprotein B mRNA editing enzyme, catalytic polypeptide-like 3A (*APOBEC3A*) was significantly altered at the transcript level [159]. This observation gains further significance considering that *APOBEC3A* is a cytidine deaminase involved in RNA editing and innate immune responses. Originally identified as a mediator of G>A editing in Wilms’Tumor 1 (*WT1*) transcripts in cord blood mononuclear cells, *APOBEC3A* has since been shown to catalyze canonical cytidine-to-uridine (C>U) RNA editing in human monocytes and macrophages, underscoring its broader involvement in innate immune regulation and transcriptomic remodeling [161]. Overall, *miR-425-5p* emerges as a candidate exosomal miRNA with potential relevance in AML pathophysiology. Therefore, beyond serving as biomarkers, AML-derived exosomes are also implicated in reshaping the bone marrow microenvironment to favor leukemic cell survival and expansion. These EVs preferentially target mesenchymal stromal and endothelial cells within the niche, where they downregulate critical hematopoietic factors such as *CXCL12*, *KITL*, *IL 7*, and *IGF 1*, while upregulating genes like *DKK1*, *IL 6*, and *CCL3* that favor leukemic cell survival and inflammation [162]. They also impair osteoblast differentiation, shifting bone marrow mesenchymal progenitors away from osteogenesis toward adipogenesis, a lineage switch contributing to bone loss and niche dysfunction [162].

#### 4.2.4. Exosomes miRNAs and Systemic Mastocytosis (SM)

Systemic mastocytosis (SM) is a rare disease that derives from an abnormal proliferation of clonal mast cells (MCs) in extracutaneous organs such as bone marrow (BM), lymph nodes, spleen, liver, gastrointestinal tract and most importantly, the skeletal system. The affected patients are at high risk of suffering from life-threatening anaphylaxis due to the massive release of mediators from mast cells and may develop even MC activation syndrome (MCAS) [163]. SM is an umbrella term which encompasses a wide spectrum of clinical entities, from indolent (ISM) and smoldering (SSM) to advanced SM (AdvSM). Advanced SM includes aggressive SM (ASM), SM with associated non-mast cell hematologic neoplasm (SM-AHN), and mast cell leukemia (MCL), which has a median OS of 2 months. The eventual associated hematologic neoplasms (AHNs) derive from the myeloid lineage in 80–90% of cases. The most common mutations detected in SM are somatic activating mutations in *cKIT*, most notably *cKITD816V*. These mutations can also be detected in the concomitant myeloid (but not in lymphoid) AHNs. Moreover, most patients with AdvSM show additional somatic mutations both on SM and AHN cells (most frequently *SRSF2*, *ASXL1*, *RUNX1 [S/A/R]*, *TET2*, and *JAK2*). This mutational multilineage involvement strengthens the hypothesis of a clonal relationship between these two diseases [164]. As mentioned above, bone involvement is one of the most frequent manifestations, characterized by both sclerotic and lytic lesions and an increased risk of pathological fractures. In this regard, a study by Kim et al. revealed a novel mechanism underlying bone disease in SM, identifying small, low-density EVs released by neoplastic MCs as active regulators of osteogenesis. These EVs are enriched in *miR-23a* and *miR-30a*, which are efficiently transferred into pre-osteoblasts, where they inhibit differentiation and bone formation. Specifically, these miRNAs downregulate key transcriptional regulators such as *RUNX2*, *SMAD1*, and *SMAD5*, thereby disrupting the bone morphogenetic protein (BMP) signaling pathway and blocking osteoblast maturation. Furthermore, by silencing these 2 miRNAs alone, the study proved that it was possible to reverse EV-induced osteogenic impairment both in vitro and in vivo. Importantly, this mechanism appears to affect bone formation independently of osteoclastogenesis, as no significant alterations in osteoclast differentiation or activity were observed [165]. Therefore, these findings suggest that MCs-derived exosomal miRNAs, particularly *miR-23a* and *miR-30a*, may serve as potential therapeutic targets for preventing or reversing bone loss in affected patients. On the other hand, further studies should be carried out to explore the role of exosomal miRNAs in the pathogenesis of SM and eventually associated hematologic neoplasms.

#### 4.2.5. Exosomes miRNAs and BCR-ABL Negative Myeloproliferative Neoplasms (MPNs)

BCR-ABL negative myeloproliferative neoplasms (MPNs) include polycythemia vera (PV), essential thrombocythemia (ET), and Primary Myelofibrosis (PMF), collectively termed classic Philadelphia-negative (Ph-) MPNs. A major breakthrough in understanding their pathobiology was the discovery of *JAK2V617F* mutation. *JAK2* regulates myelopoiesis via signaling through receptors such as erythropoietin (EPO), thrombopoietin (TPO), and G-CSF receptors. Nearly all PV patients and a significant proportion of ET and PMF patients harbor *JAK2* mutations, leading to constitutive activation of the JAK/STAT pathway and cytokine-independent cell proliferation. Additional mutations in *MPL*, *LNK*, *CBL*, *TET2*, *ASXL1*, *IDH*, *IKZF1*, *EZH2*, *DNMT3A*, *TP53*, and *SF3B1* contribute to disease heterogeneity [166]. PV arises from mutated multipotent stem cells capable of erythroid colony formation without exogenous EPO. Clonal proliferation of myeloid precursors leads to erythrocytosis often coupled with leukocytosis and thrombocytosis. MicroRNAs such as *miR-451* and *miR-144*, transcriptionally regulated by *GATA1*, are consistently upregulated in myeloproliferative neoplasms (MPNs) and play a crucial role in promoting erythroid maturation [167,168]. Mechanistically, the *miR-144/451* cluster functions as a post-transcriptional regulatory unit that modulates several essential processes during red blood cell development. The first miRNA, *miR-451*, targets *MYC*, thereby reducing proliferation and enabling terminal erythroid differentiation. It also represses *COX10*, regulating mitochondrial oxidative phosphorylation, reducing reactive oxygen species (ROS) levels and preventing oxidative stress in maturing erythroblasts. The second miRNA, *miR-144,* suppresses *RAB14* and *CAP1*, coordinating vesicle trafficking and actin cytoskeleton remodeling (two processes essential for enucleation and final maturation of erythroid cells) [167]. Conversely, *miR-150* is frequently downregulated in MPNs, a change that contributes to hematopoietic dysregulation. Under physiological conditions, *miR-150* targets the transcription factor *c-MYB*. High *miR-150* levels promote megakaryocytic differentiation at the expense of erythroid lineage commitment. Therefore, its downregulation directs differentiation towards erythroid and myeloid expansion, which may contribute to the erythrocytosis and leukocytosis observed in disorders like polycythemia vera (PV). The aberrant expression of *miR-150* is thus functionally linked to the clonal expansion of hematopoietic precursors [169].

Furthermore, overexpression of *miR-16-2* promotes erythropoiesis independently of *JAK/STAT* signaling [170]. Interestingly, some miRNAs result variably in PV according to the cell type they belong to as follows: granulocytes (let-7a downregulated, miR-182 upregulated), mononuclear cells (miR-143, miR-145 and miR-223 upregulated), and platelets (miR-26b upregulated) [171]. Among these miRNAs, *let-7* helps regulate blood cell growth by suppressing *HMGA2*, a gene that promotes cell survival. When *let-7* levels are low, *HMGA2* becomes overactive, giving mutated cells (like those with *JAK2V617F* mutation) a survival advantage [172]; *miR-223-3p* acts as a regulator of cell proliferation and apoptosis. In K562 cells, overexpression of *miR-223-3p* promotes proliferation and inhibits apoptosis by targeting and suppressing *TGFBR3* and downstream signaling molecules *Smad2/3* and *P-Smad2.* It also enhances hemoglobin production, suggesting a role in erythroid maturation [173]; *miR-145* plays a key role in erythroid differentiation, particularly in megakaryocyte-erythroid progenitor cells. Its upregulation has been observed in PV, where it contributes to the lineage commitment of hematopoietic stem/progenitor cells toward erythropoiesis, helping define the erythroid phenotype of PV [174].

Essential thrombocythemia is marked by persistent thrombocytosis, BM megakaryocytic hyperplasia, and increased thrombotic/hemorrhagic risk. *JAK2V617F* mutation is detected in about 50% of ET patients. MicroRNAs dysregulated in ET include decreased *miRs-34a*, *342*, *326*, *105*, *149*, *14,* and ectopic expression of *miRs-10a* and *150* [175]. These deregulated miRNAs play a crucial role in regulating hematopoietic proliferation and differentiation, particularly within the megakaryocytic lineage. Downregulation of *miR-34a* results in reduced apoptosis mediated by the *p53* pathway and enhanced cell survival through dysregulation of the *p53/SIRT1* axis [176]. Similarly, *miR-342*, *miR-326*, *miR-105*, *miR-149*, and *miR-147*, normally involved in controlling proliferative and inflammatory responses via inhibition of pathways such as *NOTCH* and *PI3K/AKT*, are decreased, creating a pro-myeloproliferative environment [177]. In contrast, ectopic overexpression of *miR-10a* and *miR-150* contributes to clonal megakaryocytic expansion. On one hand, *miR-10a* modulates post-transcriptional regulation of key genes in megakaryocytic development and reduces differentiation by suppressing GP Ibα, a key component of the platelet glycoprotein complex necessary for mature megakaryocyte function [178]; on the other hand, *miR-150* targets the oncogene *c-Myb* (as previously mentioned), directing CD34+ hematopoietic progenitors toward the megakaryocytic lineage at the expense of erythroid differentiation [169]. Collectively, these miRNA alterations promote megakaryocytic hyperplasia, persistent thrombocytosis, and increased thrombotic risk. Lastly, downregulation of *miRs-133a* contributes to increased hematopoietic proliferation in both PV and ET [179]. More specifically, in neutrophils from patients with polycythemia vera (PV) and essential thrombocythemia (ET), expression profiling revealed significant downregulation of *miR-133a* compared to healthy controls [179]. This microRNA is recognized as a tumor suppressor and regulator of cellular proliferation and differentiation. Functionally, it represses genes such as *cyclin D1* and signaling mediators within the *ERK1/2* pathway, thereby enforcing cell cycle checkpoints and preventing unwarranted cell division in physiological conditions. Thus, loss of *miR-133a* expression in these diseases leads to unchecked expansion of hematopoietic precursors [180], facilitating increased myeloid, and erythroid progenitor proliferation. In Primary Myelofibrosis, abnormal megakaryopoiesis triggers marrow fibrosis, osteosclerosis, angiogenesis, and extramedullary hematopoiesis. Several miRNAs, namely *miR-31*, *miR-150*, and *miR-95* are significantly downregulated in PMF. Loss of *miR-150* drives to disrupted megakaryocyte differentiation, megakaryocyte expansion, altered fibrotic signaling control, and pro fibrotic cytokine release; *miR-31* and *miR-95* regulate elements of cell cycle progression and *PI3K/AKT-TGF-β* signaling, and their reduction enhances proliferation and fibrogenesis [181]. On the other hand, *miR-190* is upregulated and may potentiate fibrotic pathways by suppressing anti-inflammatory or anti-fibrotic targets (a mechanism not yest completely elucidated) [181]. Progression from PMF to secondary AML is associated with downregulation of *miR-4319* and upregulation of *SETBP1*, a known oncogene in myeloid malignancies. Interestingly, *miR-4319* may act as a tumor suppressor miRNA embedded within the *SETBP1* gene. Its downregulation may relieve repression of *SETBP1*, thus facilitating PMF leukemic transformation [182].

All key exosomal microRNAs listed in hematological malignancies of the myeloid lineage described in the upper sections are summed up in Table 2.

### 4.3. Exosomal miRNAs and Late-Onset Acute Graft Versus Host Disease (LA-GVHD)

Late-onset acute graft-versus-host disease (LA GVHD) is a clinically significant complication following allogeneic hematopoietic stem cell transplantation (Allo-HSCT) whose underlying pathophysiology remains incompletely characterized. Though not representing a hematologic neoplasm, a special mention should be made about this particular condition since in many cases hematologic diseases could be potentially eradicated only with an Allo-HSCT [183]. Advances in molecular diagnostics have underscored the potential of exosomal miRNAs as non-invasive biomarkers even in this context. In a recent investigation, exosomal miRNA profiles were compared across patients with LA GVHD, non-GVHD transplant recipients, and healthy controls. Fifty-five miRNAs exhibited differential expressions, of which ten, most notably *miR-128*, demonstrated the most significant variation. Quantitative RT-PCR confirmed that exosomal *miR-128* was markedly upregulated at the onset of LA GVHD, with ROC analysis yielding an AUC of 0.975, indicating strong diagnostic potential. Moreover, longitudinal analyses revealed that *miR-128* levels rose prior to the clinical manifestation of the disease, thereby identifying it as a promising early predictive biomarker. These findings further suggest that *miR-128* may exert a functional role in the immunopathogenesis of LA GVHD, potentially through modulation of immune reconstitution and inflammatory signaling pathways (targeting genes such as *BMI1* and *FBXW7*). Interestingly, the expression profile of certain miRNAs, including *miR-423-5p* and *miR-142-3p*, parallels those observed in classic acute GVHD, suggesting shared pathogenic mechanisms rooted in donor T-cell alloreactivity [184]. The data support the notion that exosomal miRNAs may offer novel avenues not only for the early detection and monitoring of LA GVHD but also for the development of targeted therapeutic strategies.

## 5. An Overview on Mechanisms of Exosomal MiRNA Interference in the Hematologic Niche

Although previous sections have explored various mechanistic aspects of exosomal miRNAs in hematologic malignancies, the current focus is specifically directed toward their role in remodeling the BM microenvironment. To consolidate and expand upon earlier insights, this section summarizes key microenvironmental alterations induced by these vesicles. Particular attention is given to pivotal regulatory proteins involved in cell signaling, differentiation, and immune surveillance, whose expression is modulated by exosomal miRNAs. By interfering with these molecular pathways, exosomal miRNAs contribute to a permissive microenvironment that facilitates tumor progression and immune suppression.

For instance, *SHIP1* is a phosphatase that negatively regulates the *PI3K/Akt* signaling pathway, playing a crucial role in immune cell activation and survival. Downregulation of *SHIP1* in T-cells leads to impaired immune responses and promotes immune evasion in cancers. In the myeloid compartment, AML-derived exosomes enriched in *miR-155* have been shown to downregulate *SHIP1* expression in T lymphocytes, impairing their activation and fostering immune suppression [145]. Homebox D10 (*HOXD10*) is a transcription factor belonging to the *HOX* gene family that regulates cell differentiation and inhibits cell migration and invasion. Loss or suppression of *HOXD10* is associated with enhanced metastatic potential in tumors. Concurrently, *miR-10b* and *miR-125b* in AML exosomes facilitate leukemic cell invasion and chemoresistance by targeting *HOXD10* and *p53*, respectively, while promoting stromal remodeling through altered cytokine secretion profiles [156,157]. Similarly, exosomal *miR-150* from AML cells dampens NK-cells cytotoxicity and modulates monocyte differentiation, contributing to the formation of an immunosuppressive niche [145]. In MDS, *miR-196a-5p* and *miR-126-5p* are implicated in skewing mesenchymal SCs differentiation, leading to aberrant hematopoiesis and supporting leukemic stem cell persistence [135]. Within the lymphoid malignancies, CLL-derived exosomes are potent modulators of the tumor milieu. Exosomal *miR-155* enhances the expansion of MDSCs and inhibits the activity of CD8+ T-cells via *PI3K/Akt* and PD-L1 pathways, effectively blunting anti-tumor immunity [85]. Furthermore, *miR-150* packaged in exosomes targets VEGF signaling in endothelial cells, disrupting angiogenic support within the BM and lymph nodes [79]. In DLBCL, exosomal *miR-125b-5p* and *miR-99a-5p* modulate the mTOR pathway, promoting survival and resistance to apoptosis, while *miR-155* and *miR-20a* carried in lymphoma exosomes interfere with dendritic cell maturation, suppressing antigen presentation and fostering immune escape [96,97,101,102,103]. Suppressor of cytokine signaling 1 (SOCS1) is a member of the *SOCS* family that acts as a negative regulator of cytokine signaling, particularly within the *JAK/STAT* pathway. Inhibition of *SOCS1* promotes sustained inflammatory signaling and can facilitate tumor immune escape. In this regard, Hodgkin lymphoma exosomes enriched in *miR-21-5p* and *miR-155-5p* further contribute to immune evasion by suppressing *SOCS1* and amplifying Treg cell expansion, which dampens anti-tumor effector responses [91]. Factor inhibiting *HIF1* is an asparaginyl hydroxylase that suppresses the transcriptional activity of Hypoxia-Inducible Factor 1-alpha (HIF-1α), thereby inhibiting hypoxia-driven angiogenesis. Inhibition of *HIF-1* leads to stabilization of HIF-1α and promotes pro-angiogenic responses. The interplay between malignant exosomes and the BM microenvironment is further evidenced by *miR-135b*-mediated pro-angiogenic effects in MM, where hypoxia-driven exosomal signaling promotes neovascularization via *FIH-1* inhibition [185]. Lastly *AURKB* and *CCND1* are two important proteins which play a fundamental role during the mitotic cycle. More specifically, *AURKB* is a serine/threonine kinase involved in chromosome segregation and cytokinesis during mitosis, and dysregulation of *AURKB* contributes to chromosomal instability and oncogenesis; *CCND1* is a cell cycle protein that regulates the transition from G1 to S phase by activating CDK4 and CDK6. Overexpression of *CCND1* promotes uncontrolled cell proliferation in various cancers. In multiple myeloma, *let-7b* and *miR-15a-5p* target *AURKB* and *CCND1*, respectively, altering MSC proliferation and skewing osteoblastic differentiation toward a more supportive stromal niche [75]. Additionally, *miR-103a-3p* and *miR-4505* in MM exosomes exacerbate transformation risk by modulating cytokine networks that enhance tumor growth [61]. Collectively, these findings underscore how tumor-derived exosomal miRNAs subvert immune surveillance and reprogram the BM microenvironment to favor leukemic and lymphomatous progression. By mediating immune suppression [28], angiogenesis [186], and stromal reorganization, exosomal miRNAs establish a dynamic, pro-tumoral ecosystem that not only supports malignant cell survival but also contributes to resistance against conventional therapies.

## 6. Recommended Panels and Current Challenges

In 2023, Moloudizargari et al. summarized and recommended a series of exosomal miRNA panels for their potential to be used as non-invasive biomarkers in different hematological malignancies. Moreover, “Panel” study of miRNAs rather than studying a single miRNA level can improve sensitivity and accuracy in diagnosis [97]. These panels are schematized in Table 3.

However, an important matter that should be addressed is how can exosomal microRNAs panels, whose utility as biomarkers is evident, be translated into regular clinical practice tools. Several are the unresolved challenges, which can be broadly categorized into technical limitations, normalization issues, and biological heterogeneity:

- TECHNICAL VARIABILITY IN ISOLATION METHODOLOGIES: Traditional ultracentrifugation has long been regarded as the gold standard for exosome isolation. Nevertheless, it suffers from poor reproducibility and contamination due to several intrinsic limitations. Specifically, this method relies on sequential high-speed spins to pellet EVs based on their density and size, yet these conditions often cause EV aggregation and co-isolation of non-vesicular contaminants such as protein polymers, viruses, and high-density lipoproteins. These issues are particularly pronounced in complex and viscous biological fluids like plasma. Furthermore, the intense centrifugal forces can damage vesicle integrity, potentially altering their morphology and biological activity. These limitations, along with time-consuming protocols and the requirement for expensive instrumentation, hinder the scalability of ultracentrifugation for routine clinical use or high-throughput applications [187]. Microfluidic platforms are miniaturized systems that manipulate fluids within chips containing microscopic channels and are used in exosome and miRNA research to rapidly isolate exosomes from small biological samples such as blood, plasma, or saliva [187,188]. In the context of exosome and miRNA research, these platforms provide a promising alternative to traditional isolation techniques. One of the most advanced modalities is acoustic-based microfluidic separation, which employs ultrasonic standing waves to exert differential acoustic radiation forces on particles based on their size, density, and compressibility. These devices typically consist of two modular zones: the first removes larger components (>1 µm), including cells and debris, while the second isolates EVs by filtering out larger microvesicles and apoptotic bodies, thereby enriching for exosomes (<200 nm). Moreover, the cutoff size for separation can be dynamically adjusted to achieve precise discrimination between EV subtypes. However, challenges remain, including limited standardization across platforms, which can affect reproducibility and downstream molecular profiling [187]. One major issue is the lack of standardization in chip design and materials, such as polydimethylsiloxane (PDMS), glass, or plastic, which affects how efficiently exosomes are captured from different bodily fluids. In addition, key settings like flow rate, electric or acoustic field strength, and channel size are often operator-dependent, making results difficult to reproduce across different laboratories. These design differences can also lead to the isolation of different EV types (e.g., more microvesicles vs. fewer exosomes), which alter downstream analyses like miRNA or protein profiling. Additionally, without agreed standards for measuring exosome purity or concentration, it is hard to apply these platforms consistently in clinical settings [189]. Furthermore, by analyzing the lipidomic and proteomic cargo of isolated exosomes, it results in a certain heterogeneity in exosomal cargo according to the isolation technique. For instance, techniques such as ultracentrifugation, size exclusion chromatography (SEC), and ultrafiltration differ significantly in their ability to retain or remove contaminating proteins, lipoproteins, and soluble factors. Ultracentrifugation often co-isolates protein aggregates, while SEC offers improved purity but may lose smaller vesicles or underrepresent certain subpopulations. This technical variability contributes to inconsistencies in downstream proteomic and lipidomic profiling, as certain proteins or lipids may be enriched or depleted depending on the method used [190].
- LACK OF A CONSENSUS ON EXOSOME CHARACTERIZATION STRATEGIES: Techniques such as Transmission Electron Microscopy (TEM) and Cryogenic Electron Microscopy (Cryo-EM) provide high-resolution imaging of exosomal morphology, yet they are limited by artifacts from sample preparation and low throughput; nanoparticle Tracking Analysis (NTA) and Tunable Resistive Pulse Sensing (TRPS) allow quantification of particle size and concentration but struggle to distinguish exosomes from contaminants. Surface plasmon resonance (SPR) and Surface-Enhanced Raman Spectroscopy (SERS) enable label-free, real-time cargo profiling but require sophisticated nanotechnology expertise. Flow Cytometry (FCM), especially when combined with imaging (Imaging Flow Cytometry, IFCM), facilitates multiparametric surface marker analysis but is hampered by sensitivity limitations for submicron particles. Asymmetric Flow Field-Flow Fractionation (AF4) has shown promise in distinguishing exosomal subpopulations but remains a niche technology [191]. At present, no single method fulfills all those critical criteria (high purity, yield, reproducibility, scalability, and affordability) needed for robust clinical employment.
- INTER-PATIENT VARIABILITY IN EXOSOMAL MIRNA EXPRESSION. Intrinsic interindividual variability and disease-independent factors can both impair the accuracy in the interpretation of circulating miRNAs and challenges efforts to establish universal reference ranges [192]. As widely explained in previous sections, the first cause of this variability is the tumor itself (and associated inflammation and immune deregulation) since miRNAs represent a specific neoplastic signature. In other words, they vary according to molecular subtypes and tumor features (e.g., oncogene overexpression, etc.). Secondly, specific signatures of circulating miRNA have also been associated with a variety of pathological conditions which can coexist with tumor as comorbidities, such as cardiovascular diseases, diabetes, liver pathologies, and sepsis [193]. In addition, other factors can influence the diversity of miRNAs levels in circulation: race, gender, lifestyle, drug assumption, smoking habits, diet, and physical activity. However, there are other variables which are more difficult to verify such as polymorphisms in miRNAs chromosome loci. An example is represented by copy number variations (CNVs) occurring in coding regions of the genome. As a result, they can deregulate certain miRNAs, alter their expression, and thus, contribute to the development of the disease [192]. Interestingly, even diet represents a variable. Several dietary constituents (resveratrol, curcumin, isoflavones, catechins, indoles, vitamins A and D) play a certain role in affecting miRNAs expression profile [194]. The rationale of the effect exerted by these substances may depend on homeostatic changes in circulating miRNA-containing vehicles (including exosomes) [195]. In addition to these considerations, it should be said that the amount of circulating miRNA may vary in the same patient over time. For example, it can be influenced by common medications (aspirin has shown to reduce *miR-126* levels). Therefore, miRNAs appear as potentially useful parameters in pharmacodynamic studies [192].
- NORMALIZATION OF EXOSOMAL MIRNA QUANTIFICATION: A major translational barrier in exosome-based diagnostics is the lack of standardized normalization strategies for exosomal miRNA quantification. Although qRT-PCR remains the gold standard for miRNA detection due to its sensitivity and specificity, its reliability is highly dependent on reference miRNAs whose expression is stable across different biological conditions, disease states, and technical protocols. Current reference small RNAs such as *U6* or *RNU44*, traditionally used in cellular RNA studies, have shown inconsistent expression in serum- or plasma-derived exosomes, particularly in pathological contexts like cancer or inflammation [196,197]. In an important effort to address this gap, Damanti et al. performed a systematic assessment of RNA-seq datasets and identified *miR-26a-5p* and *miR-486-5p* as promising endogenous reference candidates in pediatric hematological malignancies. Their validation across diverse disease subtypes of lymphomas and B-cell ALL demonstrated superior stability of *miR-26a-5p*, independent of disease status or exosome isolation method (ultracentrifugation vs. kit-based protocols). This interesting data positions *miR-26a-5p* as a bona fide universal calibrator for plasma exosomal miRNA studies. Conversely, *miR-486-5p*, while abundant and stable across disease groups, was highly susceptible to the choice of isolation technique, showing significant variation between protocols [15]. This observation is particularly relevant in multi-center or retrospective studies, where differences in isolation methods (e.g., ultracentrifugation, SEC, precipitation kits) are common and often unavoidable (as previously discussed). Therefore, using normalization controls that are method-sensitive could bias miRNA levels, leading to false-positive/negative biomarker signals. Critically, several issues remain unresolved as follows: (1) this study is limited to pediatric lymphoid malignancies and extrapolation to adult cohorts or myeloid neoplasms remains speculative; (2) the identified normalizers may not generalize across biofluid types (e.g., urine, CSF) or technical platforms (e.g., digital PCR, NGS). On a final analysis, the study provides valuable empirical evidence supporting *miR-26a-5p* as a technical normalizer, but broader standardization efforts (including reference selection) are still needed.

**Figure 3:**
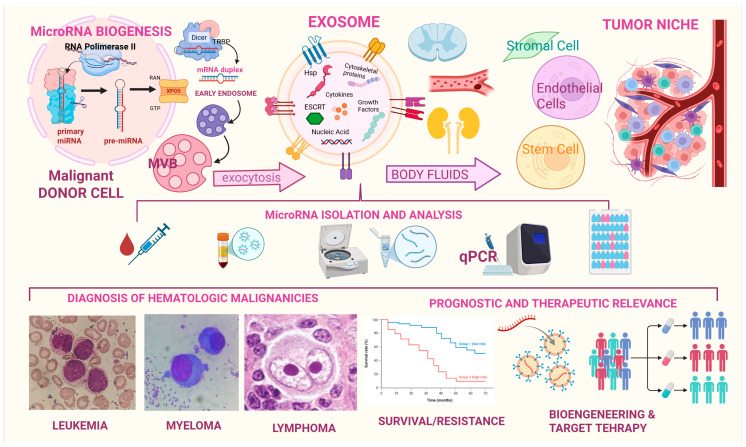
This image depicts exosome biogenesis from malignant hematologic cells, the selective packaging and release of miRNAs, their circulation through bodily fluids, and subsequent uptake by recipient cells, the workflow of exosomal microRNA isolation and analysis, their proposed role as biomarkers for diagnosis and prognosis, and their potential use to develop a target therapy in hematologic malignancies.

It is evident that the transition from exploratory studies to clinical-grade assays requires rigorous validation across diverse populations and harmonization of technical protocols. Addressing these barriers demands a coordinated effort to develop universally accepted isolation protocols, robust normalization strategies, and multi-institutional validation pipelines. Achieving a final standardization represents a fundamental passage toward the establishment of exosome-based liquid biopsy in hematological malignancies, offering a non-invasive avenue for diagnosis, prognosis, and treatment monitoring (Figure 3).

## 7. Recent Advances

In the previous section, major technical limitations have been described. Fortunately, recent advances in exosome detection and isolation techniques are directly tackling critical challenges of purity, reproducibility, and scalability. Furthermore, recent studies on exosome cargo enrichment strategies and further understandings of exosome biology are promising to improve diagnostic precision and disease monitoring. Most of the results come from studies on solid tumors.

- Ubiquitous tetraspanins such as CD9, CD63, and CD81 remain gold-standard markers used in affinity capture workflows, given their abundant expression on exosomal membranes across cell types. These membrane proteins are known to possess multiple functional roles in several biological processes, such as cell adhesion, fusion, signaling, and trafficking [198]. Apart from these classical markers, surface antigens such as epithelial cell adhesion molecule (EpCAM), EGFR, integrins (e.g., α6β4, αvβ5), and even PD-L1 have been used for immunoaffinity capture of tumor-derived exosomes, significantly improving specificity and reducing contamination from non-vesicular particles or lipoproteins [199]. A recent study introduced the “EVs on Demand” (EVOD) chip, designed to selectively capture cancer-related exosome subpopulations. The chip uses a chemical reaction between tetrazine-tagged antibodies (targeting EpCAM and EGFR) and a specially coated microfluidic surface to isolate exosomes. It successfully captured 76% more EGFR-positive exosomes from cancer patients compared to healthy individuals. However, this approach is still limited by high costs [200].
- Magnetic bead-based immunoaffinity enrichment is another separation method that has recently gained attention. This approach employs antibody-modified magnetic beads to capture exosomes. In a later passage, exosomes are separated by magnetic force. Furthermore, novel immuno-affinitive superparamagnetic nanoparticles (IS-NPs) have shown higher yield and increased purity than conventional separation methods. For example, in a study by Fang et al., superparamagnetic nanoparticles were combined with anti-CD63 antibodies through a molecular interaction between β-cyclodextrin (β-CD, a heptasaccharide derived from glucose) and 4-aminoazobenzene (AAB, an aromatic amine). This system achieved impressive results, with exosome capture and release efficiencies reaching 80% and 86.5%, respectively, in artificial model sample [201].
- Lipid-based separation techniques exploit the natural structure of exosome membranes, which are made of lipid bilayers. These lipids can interact with specially designed molecules to help isolate exosomes efficiently. In a study, Wan et al. created a special probe called a lipid nanoprobe to quickly extract exosomes from plasma. This probe includes a molecule called DSPE-PEG-biotin. More specifically, DSPE is a lipid that can insert itself into the exosome’s membrane through hydrophobic interactions; polyethylene glycol (PEG) is attached to DSPE to make the molecule soluble in water, preventing aggregation. Once the probe embeds into the exosome membrane, the biotin on its surface binds strongly to NeutrAvidin, which is coated on magnetic beads [202]. This interaction allows the exosomes to be pulled out quickly (just 15 min) and efficiently using a magnet, a much faster process than traditional isolation techniques.
- Advanced microchip technologies such as nanoplasmonic exosome assay (nPLEX) have significantly improved the sensitivity and speed of exosome detection. This technology employs nanohole arrays embedded in a thin gold film, which are functionalized with antibodies targeting exosome surface markers (e.g., CD63 and EpCAM). The metal nanohole array supports surface plasmon resonance (SPR), a phenomenon where the light energy provided by laser or LED causes electrons on the metal surface to oscillate in resonance. These oscillations are sensitive to changes on the surface of the array, such as when exosomes bind to the antibodies anchored within the holes. The binding causes a shift in the resonance signal (e.g., change in transmitted light intensity or wavelength), which is measured by a sensor. By monitoring these optical changes, scientists can detect and quantify very small amounts of target molecules (thousands/µL) without the need to attach any additional markers (like fluorescent dyes, radioactive isotopes, or enzymes) to the particles they are trying to detect [203].
- A novel digital microfluidic (DMF) platform was developed for automated, rapid, and low-volume EVs pretreatment. The system combines a reusable DMF chip with a magnetic particle-based protocol, enabling complete exosome isolation and miRNA extraction within 20–30 min from as little as 20–40 μL of plasma. This DMF system operates by manipulating droplets on an electrode array chip, allowing for precise, programmable control of EVs isolation, washing, and lysis steps. The method was validated using clinical plasma samples from patients with non-small cell lung cancer (NSCLC). RT-qPCR analysis revealed that EV-derived *miR-486-5p* and *miR-21-5p* were effective biomarkers for NSCLC, and the results were consistent with those obtained using a commercial exosome RNA extraction kit. Importantly, the platform achieves over 77% isolation efficiency and is cost-effective due to chip reusability. This advance demonstrates strong potential for standardized, scalable EV-miRNA–based liquid biopsy applications in cancer diagnostics [204].
- Droplet digital PCR (ddPCR) is a highly sensitive technique that improves nucleic acid detection by dividing the PCR reaction mixture into thousands of tiny droplets. Typically, each droplet contains either zero or one copy of the target gene. After PCR, droplets are classified as positive or negative based on their fluorescence signal, and the concentration of the target molecule is calculated using the Poisson distribution and the ratio of positive droplets. Compared to traditional qPCR, ddPCR offers greater sensitivity and accuracy, particularly for detecting low-abundance targets such as urinary exosomal miRNAs. Recent studies highlight its utility for liquid biopsy in cancer diagnostics. For example, exosomal *miR-15a-5p*, measured by ddPCR, was found to effectively distinguish endometrial cancer patients from healthy individuals [205]. Therefore, ddPCR has shown to improve sensitivity and reproducibility in miRNAs detection from limited blood volumes. For this reason, it appears particularly suited to monitoring minimal residual disease (MRD) or detecting low-expression miRNAs in blood cancers, where early relapse detection is critical [206].
- The CRISPR/Cas system, widely known for its role in gene editing, has recently been adapted for the highly sensitive and specific detection of exosomal miRNAs. A notable example is the RACE (rolling circle amplification–Assisted CRISPR/Cas9 Cleavage) method developed by Wang et al., which combines nucleic acid amplification with CRISPR/Cas-based cleavage. In this approach, a specially designed DNA padlock probe (a short single-stranded DNA molecule) binds to the target miRNA, accurately identifying even single-nucleotide differences. This probe is then circularized. This circular DNA is amplified through rolling circle amplification (RCA), producing long single-stranded DNA (ssDNA) that contains multiple repeats of the target sequence along with PAM motifs (short, specific DNA sequences) necessary for Cas9 recognition. The CRISPR/Cas9 complex then cleaves these amplified sequences. Simultaneously, a TaqMan probe is added to the reaction and binds the target DNA. When Cas9 cuts the target DNA, the probe is cleaved, producing a measurable fluorescence signal that indicates the presence of the miRNA [207]. This method is highly sensitive even for small differences in miRNA sequence and is able to detect multiple miRNAs in a single test.

Recent progress in understanding how miRNAs are selectively loaded into exosomes has identified key regulatory mechanisms, particularly the role of RNA-binding proteins (RBPs) and specific sequence motifs. These findings are essential for improving the diagnostic precision of exosomal miRNA profiling and addressing technical variability in biomarker studies. A notable example involves Synaptotagmin-binding cytoplasmic RNA-interacting protein (SYNCRIP), an RBP present in extracellular vesicles from various cell types. In a study by Santangelo et al., Western blot analysis confirmed that SYNCRIP specifically binds to miRNAs enriched in exosomes (hEXO-miRNAs), such as *miR-3470a* and *miR-142-2-3p*. These miRNAs share a conserved GGCU sequence, which was identified as the SYNCRIP recognition motif responsible for their exosomal localization [208]. Importantly, disrupting SYNCRIP expression or mutating the GGCU motif led to a significant reduction in the levels of these miRNAs within exosomes, underscoring the functional importance of this sorting mechanism. Among the latest advancements, mention should be made of exosome enrichment methodologies. A very recent proof-of-concept study investigated the feasibility of using leukemia-derived exosomes as a source of tumor-specific double-stranded DNA (dsDNA) for non-invasive biomarker detection in AML patients. Researchers employed a CE-IVD (Conformité Européenne-In Vitro Diagnostic) certified kit to enrich exosomes from patient plasma. This process was followed by NGS analyses of the exosomal dsDNA. The results revealed a strong correlation (R = 0.849) between exosomal DNA concentration and leukemia burden, with the genomic profile of exosomal DNA closely mirroring that of BM blasts (rather than PB cells). This suggests that BM-resident leukemic cells are the primary contributors to exosomal DNA, likely due to their active interaction with the BM microenvironment. The exosomal DNA contained a wide range of leukemia-associated mutations (*ASXL1*, *NRAS*, *TP53*, *RUNX1*, *CEBPA*, *FLT3*, *IDH1*, *IDH2*, *NPM1*, *DNMT3A*, *TET2*, *KRAS*, *ETV6*, *SF3B1*, *GATA2*) and remained detectable even in patients in complete remission. This data highlighted its potential for MRD monitoring and early relapse detection [209]. Despite the kit’s limitation in allowing exosome characterization, the study demonstrated that exosome-based liquid biopsy offers a sensitive and standardized approach for AML diagnosis, treatment response assessment, and relapse prediction. Therefore, it would be very interesting to replicate the same study using exosomal microRNAs as an alternative source of non-invasive tumor biomarkers.

While the majority of technological advances in exosome characterization, isolation, and molecular profiling have been developed and validated within the context of solid tumors, their underlying principles are potentially applicable even in hematology. Focused research efforts are now warranted to determine the diagnostic accuracy, reproducibility and clinical utility of such approaches in the hematological setting. Rigorous evaluation in appropriately designed studies will be essential to establish whether these methodologies can be reliably integrated into diagnosis, MRD surveillance, treatment response monitoring, and early relapse detection in patients with hematologic neoplasms.

## 8. Conclusions and Future Perspectives

Exosomes have emerged as sophisticated mediators of intercellular communication, with particular significance in the context of hematological malignancies. These extracellular vesicles, once regarded as mere cellular debris, are now recognized as dynamic entities capable of transferring a diverse array of bioactive molecules, including proteins, lipids, and, most notably, miRNAs, between cells. Among their various cargo, exosomal miRNAs have garnered considerable attention due to their remarkable stability, specificity, and biological relevance. Unlike free-circulating miRNAs, many of which are passively released by apoptotic or necrotic cells and may not accurately reflect tumor-specific biological processes, exosomal miRNAs are actively and selectively packaged and secreted. This endows them with the potential to serve as more precise molecular biomarkers. Their capacity to alter gene expression in recipient cells, facilitating processes such as angiogenesis, immune modulation, and tumor cell invasion, underscores their dual role as both biomarkers and functional drivers of disease progression. In hematological neoplasms, exosomal miRNAs hold immense promise for diagnosis, prognosis, and therapeutic monitoring, offering valuable insight into disease classification, staging, and progression. They may also aid in stratifying patients based on molecular profiles and response to therapy. Moreover, exosomes derived from BM mesenchymal stromal cells (MSCs) and leukemic blasts contribute to drug resistance, immune evasion, and the shaping of a pro-tumorigenic microenvironment, further affirming their biological and clinical relevance. Despite these promising attributes, several challenges remain before exosomal miRNAs can be fully integrated into clinical practice. Among them are the lack of standardized protocols for exosome isolation and miRNA detection, the variability of reference gene selection, and concerns related to exosome heterogeneity, half-life, scalability, and bioavailability. These technical and logistical barriers must be addressed to enable the consistent and reproducible application of exosomal miRNAs as clinical tools. Nonetheless, the field is advancing. Several engineered exosome-based therapies have entered clinical trials, marking a transition from bench to bedside. Although these developments pertain primarily to solid tumors, they exemplify the broader therapeutic potential of exosomal technology. In conclusion, exosomal miRNAs represent a compelling frontier in cancer biology, offering not only a window into the molecular architecture of hematological malignancies but also a platform for targeted therapeutic interventions. As research continues to refine the tools and methodologies necessary for their clinical deployment, exosomal miRNAs may well become integral to the personalized management of blood cancers, enabling earlier diagnosis, more accurate prognostication, and ultimately, more effective treatment strategies.

## Figures and Tables

**Table 1:** Key exosomal microRNAs in hematological malignancies of the lymphoid lineage: functions, targets, and clinical relevance. ABBREVIATIONS: MM (multiple myeloma); MGUS (monoclonal gammopathy of undetermined significance); CLL (chronic lymphoid leukemia); HL (Hodgkin lymphoma); DLBCL (Diffuse Large B-Cell Lymphoma); PFS (progression free survival); ALL (acute lymphoblastic leukemia).

Disease	Principal miRNA	Principal Biological Function	Key Targets/Pathways	Clinical Role
MM	*miR-20a-5p*	Promotes chemoresistance	*RUNX3*, *Rab27B*, *ATG7*	Early disease progression marker
	*miR-103a-3p*	Promotes progression	*PTEN/PI3k-Akt*	Biomarker of MGUS → MM shift
	*miR-4505* *miR-10a*	Enhances tumor aggressiveness Pro-oncogenic	Cytokine network *EPAH8/SEMA5A*	Prognostic marker Favors proliferation and metastasis
	*miR-16*	Regulates IGF1R signaling	*IGF1R/CCND1/ELAVL1*	Low levels linked to poor prognosis
	*PRINS (lncRNA)*	Reflects chromosomal instability	del(13)(q14), t(4;14)	Distinguishes MM from healthy donors
	*miR-135b* *miR-21*	Pro-angiogenic in hypoxic BM niche Counteract apoptosis	*FIH-1* *Rhob*	Target for anti-angiogenic therapy Induces steroid-resistance
	*miR-15a-5p*	Tumor suppressor	*CCND1*, *BCL2*	Reduced in bortezomib-resistant MM
	*miR-17-5p*	Both: tumor suppressor/onco-miR	*HBP1*, *AIB1*, *E2F1*	Reduced in bortezomib-resistant MM
	*miR-18a*	Drives extramedullary disease	*Dicer*, *HIF-1α*	Marker of aggressive disease
CLL	*miR-15a*, *miR-16*	Induce apoptosis	*BCL2*	Loss correlates with poor prognosis
	*miR-155*	Promotes MDSCs	*PI3K/Akt*, *PD-L1*	Marker of immune escape/resistance
	*miR-150*	Regulates T-cell differentiation	*MYB*, *NOTCH*	Prognostic marker/identifies CLL
	*miR-223*	Immune suppression	*STAT3*	Downregulated in case of progression
	*miR-202-3p*	Activates Hedgehog pathway	*SMO*	Marker for immune escape
	*miR-195*	Correlates with time-to-treatment	*CCND1*	Early detection biomarker
HL	*miR-24-3p*	Regulates cell cycle	*CDK6*, *SMAD4*	Diagnostic/reduced after treatment
	*miR-127-3p*	Regulates cell cycle	*CDK6*, *SMAD4*	Diagnostic/reduced after treatment
	*miR-21-5p*	Tumor growth/immune evasion	*PTEN*, *SOCS1*	Marker for disease activity and relapse
	*miR-155-5p*	Tumor growth/immune evasion	*PTEN*, *SOCS1*	Marker for disease activity and relapse
	*Let-7a-5p*	Tumor suppressor	*RAS*, *HMGA2*	Monitoring disease response
DLBCL	*miR-379-5p*	Promotes survival/chemoresistance	*BCL2*, *LIN28B*	Early diagnostic marker
	*miR-135a-3p*	Promotes survival/chemoresistance	*BCL2*, *LIN28B*	Early diagnostic marker
	*miR-99a-5p*	Negatively targets apoptosis	*mTOR*, *IGF-1R*	Marker of chemoresistance
	*miR-125b-5p*	Negatively targets apoptosis	*mTOR*, *IGF-1R*	Predicts shorter PFS
	*miR-451a*	Suppresses invasion	*MIF*	Low levels linked to poor prognosis
	*miR-155*	Regulates immune cell function	*SHIP1*, *PU.1*	Marker of refractory-relapsed disease
	*miR-20a*	Regulates immune cell function	*SHIP1*, *PU.1*	Associated with higher mortality rate
	*miR-106a/b*	Enhances cell survival and invasion	*E2F1*, *TGFβ*	Associated with higher mortality rate
ALL	*miR-181a*	Induces CNS involvement	*MCL-1*, *BCL2*	CNS relapse biomarker
	*miR-181b-5p*	Suppresses apoptosis	*PCNA*, *Ki-67*	Potential therapeutic target

**Table 2:** Key exosomal microRNAs in hematological malignancies of the myeloid lineage: functions, targets, and clinical relevance. ABBREVIATIONS: CML (chronic myeloid leukemia); VEGF (vascular endothelial growth factor); MDS (myelodysplastic syndromes); AZA (azacitidine); LENA (lenalidomide); AML (acute myeloid leukemia); OS (overall survival); SM (Systemic Mastocytosis); PV (polycythemia vera); ET (essential thrombocythemia); PMF (Primary Myelofibrosis).

Disease	Principal miRNA	Principal Biological Function	Key Targets/Pathways	Clinical Role
CML	*miR-92a*	Modulates angiogenesis	Integrin α5	Biomarker for vascular remodeling
	*miR-210*	Promotes VEGF-mediated angiogenesis	*EPHRIN-A3*	Potential therapeutic target
	*miR-126*	Affects adhesion/migration of CML cells	*CXCL12*, *VCAM1*	Modulator of leukemic niche
MDS	*miR-196a-5p*	Regulates hematopoiesis/therapy resistance	*HOXA*, *DNMT1*, *PTEN*	Associated with progression to AML
	*miR-126-5p*	Regulates hematopoiesis/therapy resistance	*PTTG3P*	Diagnostic/predictive of AZA response
	*miR-192-5p* *let-7a* *mir-196-5b*	Targets BCL2 and suppresses proliferation Associated with higher-risk karyotype Drives aberrant proliferation	*BCL2* *RAS/MYC* *CDKN1B*	Predictor of AZA/LENA therapy success Favors proliferation Associated with progression to AML
AML	*miR-150*	Modulate apoptosis and proliferation	*PKCα*, *FOXO4*	Early detection marker
	*miR-155*	Promotes leukemogenesis	*SHIP1/SOCS1*	Early detection marker
	*miR-10b*	Enhances tumor invasiveness	*HOXD10*, *RhoC*	Poor prognosis biomarker (shorter OS)
	*miR-125b*	Promotes chemoresistance Inhibits differentiation	*p53*, *BAK1*, *CBFβ* *TET2*	Associated with relapse and mortality Associated with relapse and mortality
	*miR-532*	Improves overall survival	Unknown	Positive prognostic marker
	*miR-1246* *miR-425-5p*	Induces survival and colony formation Tumor suppressor	*LRIG1*; *JAK/STAT* *23 genes* (e.g., *APOBEC3A*)	Detection/aggressive disease marker Poor prognosis when deregulated
SM	*miR-23a*	Inhibits osteogenesis	*RUNX2*, *SMAD1*, *SMAD5*	Potential targets for reversing bone loss
	*miR-30a*	Same as above	Same as above	Same as above
PV	*miR-451*, *144*	Promote erythroid maturation	*MYC/COX10*; *RAB14/CAP1*	Enhance erythropoiesis
	*miR-150*	Suppresses erythroid differentiation	*c-MYB* inhibition	Contributes to clonal expansion
	*miR-16-2*	Promotes erythropoiesis	(Independent of *JAK/STAT*)	Supports erythroid colony formation
	*let-7a*, *miR-182*	Regulate hematopoietic proliferation	*PI3K/AKT*	Their dysregulation alters hematopoiesis
	*miR-143*, *145*, *223*	Modulate platelet differentiation	*PI3K/AKT*	Contribute to aberrant hematopoiesis
ET	*miR-34a*	Induces apoptosis via p53/SIRT1 axis	p53/SIRT1	Loss favors megakaryocytic hyperplasia
	*miR-342*, *326*	Regulate inflammatory responses	*NOTCH*, *PI3K/AKT*	Loss favors meyeloproliferation
	*miR-105*, *149*, *147*	Modulate inflammatory pathways	*PI3K/AKT*, *NF-κB*	Dysregulation promotes proliferation
	*miR-10a*	Regulate megakaryocyte development	*MPL/JAK2*	Favors megakaryocytic expansion
	*miR-150* *miR-133a*	Favors megakaryocytic lineage Tumor suppressor/regulate differentiation	*c-MYB* inhibition *Cyclin D1*; *ERK1/2*	Thrombocytosis and risk of thrombosis Downregulation leads to proliferation
PMF	*miR-31*, *150*, *95*	Downregulated: impairs differentiation	Myb; *PI3K/AKT-TGFβ*	Associated with marrow fibrosis
	*miR-190*	Upregulated: pro-survival function	Unknown	Cellular persistence in fibrotic BM
	*miR-223*, *146b*	Regulate megakaryocyte proliferation	*NF-κB*, *JAK/STAT*	Contribute to fibrosis and splenomegaly
	*miR-4319*	Downregulated: SETBP1 upregulation	*SETBP1* pathway	Associated with progression to AML

**Table 3:** Recently recommended miRNA panels that can be employed to better characterize major onco-hematologic neoplasms. ABBREVIATIONS: AML (acute myeloid leukemia); CML (chronic myeloid leukemia); ALL (acute lymphoblastic leukemia); MDS (myelodysplastic syndromes); PI3K (Phosphoinositide 3-Kinase)/AKT (Protein Kinase B); MM (multiple myeloma); TGF (transforming growth factor); CHT (chemotherapy); EMD (extramedullary disease); NDMM (newly diagnosed MM); miRNA (micro-RNA); WM (Waldenström macroglobulinemia); DLBCL (diffuse large B-cell lymphoma).

Disease	Micro-RNA	Main Function	Key Targets	Role as Biomarker
AML	*miR-150*	Modulates apoptosis, differentiation, tumor growth	*PKCα*, *FOXO4*, *iASSP*,	Distinguishes AML from CML, ALL, MDS
			*EIF4B*, *TET3*	
	*miR-155*	Regulates proliferation and apoptosis via PI3K/Akt signaling	*PU.1*, *SHIP1*	Indicates progression/refractoriness
	*miR-1246*	Promotes angiogenesis and tumor aggressiveness	*PML*, *ALDH1*, *SOX2*	Indicates resistance/invasiveness
MM	*miR-17-5p*	Tumor suppressor and oncogene (context-dependent)	*HBP1*, *AIB1*, *E2F1*	Reduced in bortezomib-resistant MM
	*miR-20a-5p*	Promotes proliferation, chemoresistance	*RUNX3*, *Rab27B*,	Reduced in bortezomib-resistant MM
			*Smad4*, *ATG7*	
	*miR-15a-5p*	Tumor suppressor, regulates apoptosis	*CCND1*, *BCL2*,	Associated with CHT resistance
			*BDNF*, *CXCL10*	
	*miR-16-5p*	Tumor suppressor, modulates TGF-β	*Smad3*	Associated with CHT resistance
	*let-7b*	Tumor suppressor, regulates cell cycle and resistance	*CCND1*, *MTDH*, *CALU*,	Associated with poor prognosis
			*ERK*, *AURKB*	in NDMM
	*miR-18a*	Promotes growth and EMD, regulates miRNA biogenesis	*Dicer*, *HIF-1α*,	Indicates progression
			*ATXN1*, *STK4*	
WM	*miR-192-5p*	Promotes proliferation, survival, metastasis	*SEMA3A*, *XIAP*, *YY1*,	Indicates progression
			*USP1*, *PI3K/Akt*	
	*miR-320b*	Tumor suppressor, reduces migration and invasion	*c-Myc*, *CD71*, *TRIAP1*,	Downregulated in aggressive forms
			*Wnt/β-catenin*, *MMP2/9*	
	*let-7d*	Dual role: regulates RAS, stem cell aging, stromal signaling	*RAS*, *HMGA2*, *P16*,	Useful to stratify high-risk patients
			*COL3A1*, *CCL7*	
DLBCL	*miR-99a-5p*	Regulates tumor growth and survival	*SMARCA5*, *SMARC1*,	Indicates progression and useful to
			*mTOR*, *IGF-1R*, *FGFR3*	distinguish disease subtypes
	*miR-125b-5p*	Tumor suppressor but also downregulates p53	*BCL-2*, *LIN28B*, *p53*	Useful to define subtype and risk level

## Data Availability

No new data were created or analyzed in this study.
